# Physical Fitness with Exercise and GLP-1 Receptor Agonist Treatment Alone or Combined After Diet-Induced Weight Loss: A Secondary Analysis of a Randomized Controlled Trial in Adults with Obesity

**DOI:** 10.1007/s40279-025-02386-0

**Published:** 2026-01-24

**Authors:** Simon Birk Kjær Jensen, Matteo Fiorenza, Christian Rimer Juhl, Rasmus Michael Sandsdal, Emma Jensen, Søren Sonnenborg Seier, Charlotte Janus, Julie Rehné Jørgensen, Martin Bæk Blond, Jens Juul Holst, Bente Merete Stallknecht, Sten Madsbad, Thomas Bandholm, Signe Sørensen Torekov

**Affiliations:** 1 Department of Biomedical Sciences, Faculty of Health and Medical Sciences, University of Copenhagen, Blegdamsvej 3, 2200 Copenhagen N, Denmark; 2 Clinical and Translational Research, Steno Diabetes Center Copenhagen, Herlev, Denmark; 3 Novo Nordisk Foundation Center for Basic Metabolic Research, University of Copenhagen, Copenhagen, Denmark; 4 Department of Endocrinology, Copenhagen University Hospital - Amager and Hvidovre, Copenhagen, Denmark; 5 Physical Medicine and Rehabilitation Research-Copenhagen, Department of Physical and Occupational Therapy, Copenhagen University Hospital - Amager and Hvidovre, Hvidovre, Denmark; 6 Department of Clinical Research, Copenhagen University Hospital - Amager and Hvidovre, Hvidovre, Denmark; 7 Department of Orthopedic Surgery, Copenhagen University Hospital - Amager and Hvidovre, Hvidovre, Denmark; 8 Department of Clinical Medicine, University of Copenhagen, Copenhagen, Denmark

## Abstract

**Background:**

Obesity is associated with impaired physical fitness, including physical functional performance and cardiorespiratory fitness, which affect health-related quality of life and mortality.

**Objective:**

We aimed to investigate the efficacy of a moderate-to-vigorous intensity exercise program and glucagon-like peptide-1 receptor agonist treatment alone or in combination during weight maintenance for physical fitness.

**Methods:**

This is secondary analysis of a randomized controlled trial involving 193 adults with obesity (age 18–65 years, body mass index 32–43 kg/m^2^) without diabetes mellitus who completed an 8-week low-calorie diet and were subsequently randomized (1:1:1:1 ratio) to: exercise plus placebo; glucagon-like peptide-1 receptor agonist liraglutide 3 mg once-daily plus usual activity; exercise plus liraglutide combined; or placebo plus usual activity. The exercise program was a combination of group sessions (interval-based indoor cycling followed by circuit training) and individual sessions of moderate-to-vigorous intensity, designed to meet the World Health Organization recommendations on physical activity for health. Exercise adherence was measured with sports watches and heart rate monitors. Key secondary endpoints related to physical fitness were changes from randomization to the end of treatment (weeks 0–52) in: (1) physical functional performance (time to ascend and descend an 11-step stairway twice); (2) cardiorespiratory fitness (peak oxygen consumption normalized to fat-free mass); and (3) muscle strength (isometric knee extensor peak torque).

**Results:**

Participants randomized to exercise performed a median 2.65 session/week (116 min/week at 79% of maximum heart rate) with no significant difference between those who received placebo or liraglutide. Compared with liraglutide alone, the combined treatment decreased time to complete a stair climb test by 1.2 s [95% confidence interval 0.6–1.9] (corresponding to 8.6%) and improved peak oxygen consumption by 3.0 mL/min/kg fat-free mass [95% confidence interval 0.5–5.5]. Exercise alone led to similar benefits, whereas liraglutide alone did not improve physical fitness. Compared with placebo (− 7.8%), relative muscle strength (strength normalized to body weight) was higher with exercise (− 0.4%), liraglutide (+ 1.0%), and the combined treatment (+ 3.3%) because of lower weight and preserved absolute strength.

**Conclusions:**

Structured exercise combined with glucagon-like peptide-1-based obesity pharmacotherapy led to clinically meaningful improvements in physical functional performance and cardiorespiratory fitness, in contrast to pharmacotherapy alone.

**Clinical Trial Registration:**

EudraCT number, 2015-005585-32; ClinicalTrials.gov number, NCT04122716.

**Supplementary Information:**

The online version contains supplementary material available at 10.1007/s40279-025-02386-0.

## Key Points

**Table:** 

We show that combined exercise and glucagon-like peptide-1 receptor agonist treatment leads to clinically relevant improvements in physical fitness in terms of physical functional performance and cardiorespiratory fitness. Despite weight reduction, glucagon-like peptide-1 receptor agonist alone is insufficient to improve physical fitness.
Medical obesity treatment should move beyond general physical activity recommendations and incorporate structured exercise interventions delivered by qualified exercise professionals, given the demonstrated benefits on physical fitness.

## Introduction

Obesity is linked to impaired physical functional performance and cardiorespiratory fitness [1–3]. Low levels of these physical fitness constructs negatively affect health-related quality of life [4, 5] and are independent predictors of functional disability or limitations in older adulthood [6] and premature all-cause mortality [7–9]. High cardiorespiratory fitness levels largely mitigate the increased risk of cardiovascular and all-cause mortality associated with obesity [10, 11]. Accordingly, updated guidelines for obesity care now emphasize that improved physical fitness should be considered a central treatment target [12].

Obtaining and sustaining a clinically relevant weight reduction with lifestyle intervention alone is challenging [13]. Obesity pharmacotherapy may be added to facilitate weight loss and maintenance and improve obesity-related complications. Liraglutide, a glucagon-like peptide-1 receptor agonist (GLP-1 RA) approved for the treatment of obesity, induces placebo-subtracted weight loss of 3–6% [14–16]. More effective pharmacotherapies are now available, reaching weight losses exceeding 20% [17]. About 20–40% of the weight lost during pharmacotherapy consists of fat-free mass (FFM) [18], which has led to concerns about potential adverse effects on muscle strength and cardiorespiratory fitness [19, 20]. However, FFM measured by dual-energy x-ray absorptiometry (DXA), i.e., the mass of all the body’s non-fat molecules (e.g., water, organ tissue, bone, and muscle) regardless of their location [21], is not equivalent to skeletal muscle mass. Obesity pharmacotherapy has been shown to reduce muscle mass as evident by a decreased thigh muscle volume measured by magnetic resonance imaging after the incretin-based treatments liraglutide [22] and tirzepatide [23]. Nevertheless, muscle strength is more strongly associated with adverse outcomes and mortality than muscle mass [24]. To determine whether the pharmacotherapy-induced reduction in muscle mass is maladaptive (i.e., impairs muscle function) or adaptive to lower body weight with minimal functional consequence, objective functional testing is required to assess muscle strength and physical functional performance.

Weight loss obtained through low-calorie diets is associated with modest reductions in absolute strength but increases in strength relative to body weight [25]. Weight loss per se may lead to some improvements in physical functional performance; however, the addition of combined aerobic and resistance exercise produces greater benefits [26]. During calorie restriction, resistance exercise alone or combined with aerobic exercise preserves muscle mass, whereas aerobic exercise alone is less effective [27]. Among people who are physically inactive and/or have obesity, aerobic exercise interventions consistently improve cardiorespiratory fitness to levels associated with substantial reductions in all-cause mortality [7, 28]. A recent meta-analysis showed that obesity accompanied by high levels of cardiorespiratory fitness was not associated with increased risk of all-cause mortality or cardiovascular disease [10]. In contrast, both obesity and normal weight, accompanied by low cardiorespiratory fitness, did show an increased risk, highlighting why cardiorespiratory fitness should be a focus in obesity management.

Obesity pharmacotherapies are approved as adjunctive treatments to increased physical activity and reduced energy intake. Still, interactions between pharmacological and non-pharmacological obesity treatment approaches remain an underexplored area of research. The aim of this secondary analysis was to investigate the efficacy of exercise and GLP-1RA treatment alone or combined during weight maintenance on objectively measured physical fitness.

## Methods

### Trial Design

This is a secondary trial report on secondary outcomes from the S-LiTE trial (EudraCT number, 2015-005585-32; ClinicalTrials.gov number, NCT04122716). The trial design is detailed in the primary trial report [29] and protocol [30]. S-LiTE was a randomized placebo-controlled trial with an initial weight loss phase before randomization. The trial was approved by the Regional Ethics Committee for the Capital Region of Denmark (H-16027082) and the Danish Medicines Agency. The trial was conducted at the Department of Biomedical Sciences, University of Copenhagen, Denmark and the Department of Endocrinology, Copenhagen University Hospital—Amager and Hvidovre, Denmark.

### Participants

Eligible participants were adults (age 18–65 years) with obesity (body mass index 32–43 kg/m^2^) and no serious chronic illnesses. Inclusion and exclusion criteria are available in the published protocol [30]. Participants were recruited through local newspapers, online media, and flyers at the Department of Endocrinology, Hvidovre Hospital and the Department of Biomedical Sciences, University of Copenhagen.

### Initial Weight Loss and Randomization

Initially, participants completed an 8-week low-calorie diet, which consisted of four meal replacement products per day (approximately 800 kcal/day, 15 g of fat, 110 g of carbohydrates, 56 g of protein). A weight loss of at least 5% of initial body weight was a criterion for randomization. After the initial diet-induced weight loss, participants were randomized (1:1:1:1) to one of four treatment arms: placebo + usual physical activity (placebo), placebo + exercise (exercise alone), liraglutide + usual physical activity (liraglutide alone), or liraglutide + exercise (combined treatment). Randomization was stratified by sex assigned at birth (male/female) and age (< 40 years/ ≥ 40 years). A study nurse, who was not blinded to study medication and not involved in any other aspect of the study, performed randomization using an allocation list provided by Novo Nordisk A/S. The study nurse did not meet the participants and provided the group allocation to the study personnel when a participant was to be randomized. The randomization list and the list used to allocate medication based on the randomization (total dispensing unit number list) were securely stored with access restricted to the study nurse. Study participants and personnel were blinded to study medication allocation until the primary endpoint analysis (change in body weight) was completed. The secondary analyses presented in this paper were not performed blinded, and we consider them exploratory. After randomization, the low-calorie diet was gradually phased out with three meal replacement products and one regular meal the first week, followed by two meal replacement products and two regular meals for 3 weeks.

### Interventions

#### Exercise Program

A detailed exercise protocol, in accordance with the CERT reporting checklist, is available in the Methods D of the Electronic Supplementary Material (ESM) in the primary trial report [29], including specific examples of exercise programs. The 52-week exercise program included a 6-week ramp-up phase, and from weeks 7 to 52, the aim was to follow the World Health Organization’s recommendations on physical activity for health, i.e., at least 150 min/week of moderate intensity, 75 min/week of vigorous intensity, or an equivalent combination of both [31]. To achieve this, participants were encouraged to perform twice-weekly supervised group sessions and twice-weekly individual sessions. Group sessions consisted of 30 min of indoor cycling followed by 15 min of circuit training. The indoor cycling was interval based, and the aim was to achieve an average heart rate of at least 80% of the maximal heart rate. The following circuit training typically consisted of three circuits with five exercises performed for 40 s interspersed with 20-s breaks. The types of exercise performed in circuit training was a combination of vigorous-intensity aerobic exercises (e.g., elliptical training, rowing, running/brisk walking, arm cranking, step exercises, boxing on bag) and muscle-strengthening exercises using own body weight or external resistance (e.g., squats, lunges, glute bridges, sit‐ups, plank, push‐ups, chest press and rows in suspension trainer, kettlebell swings). The type of exercise performed at individual sessions was of own choice but guided by the study staff, and was encouraged to be of moderate-to-vigorous intensity. The most frequently used exercise modalities for individual sessions were cycling, running, brisk walking, and circuit training. Heart rate monitors were used to monitor all group and individual exercise sessions, and intensity was measured based on the maximum heart rate defined as the highest value obtained during the cycle tests to exhaustion performed before and after the low-calorie diet: light intensity (50–63% of maximum heart rate), moderate intensity (64–76%), and vigorous intensity (≥ 77%). The exercise intervention was flexible in the sense that, in agreement with study personnel, if deemed necessary to improve adherence, participants could substitute group exercise with individual exercise and/or reduce exercise frequency if the duration of exercise sessions was increased correspondingly. Participants who were not randomized to exercise were instructed to maintain their usual physical activity.

#### Study Medication

Liraglutide or volume-matched placebo was initiated at 0.6 mg once daily with weekly increments of 0.6 mg until 3.0 mg was reached. Participants who could not tolerate 3.0 mg remained on the maximum tolerated dose in agreement with medical doctors blinded to the study medication.

#### Dietary Advice

All participants, independent of the treatment group, received 12 scheduled consultations on weight maintenance dietary advice, guided by a pamphlet provided at randomization. Consultations covered the Danish dietary guidelines, high- and low-satiety foods, practical habit-change strategies, and recommendations for maintaining weight after weight loss (for details, see Methods B of the ESM in the primary trial report [29]).

### Experimental Days

All outcomes related to physical fitness were assessed on 3 identical experimental days: at inclusion before starting the low-calorie diet (week − 8), at randomization after completing the low-calorie diet (week 0), and at the end of treatment 52 weeks after randomization (week 52). The physical fitness tests were carried out in the following order: (i) physical functional performance test; (ii) muscle strength test; and (iii) cardiorespiratory fitness test.

#### DXA

Whole-body DXA (Hologic Discovery A; Hologic Inc., Bedford, MA, USA) was performed at the beginning of the test days to measure total fat mass, total FFM, and appendicular FFM (the sum of arms and legs FFM).

#### Physical Functional Performance Test

Physical functional performance was measured as the time to ascend and descend an 11-step stairway twice as fast as possible. The stairway had a ~ 2 × 2 m platform at the top and bottom. The test began with participants standing at the bottom platform with both feet pointing towards the stairs. Participants climbed to the top platform, descended to the lower platform, and repeated once. The use of handrails for balance support was allowed if needed. The participants were given the option of one practice try before the test and were allowed to take two steps at a time.

#### Cardiorespiratory Fitness Test

Cardiorespiratory fitness was assessed using an incremental exercise test to exhaustion on an electromagnetically braked cycle ergometer (Corival; Lode Medical Technology, Groningen, The Netherlands). The test protocol consisted of two consecutive 4-min submaximal bouts (40 and 80 W for female individuals, 50 and 100 W for male individuals) followed by stepwise workload increments (20 W/min for female individuals, and 25 W/min for male individuals) until volitional exhaustion. During the incremental test, pulmonary gas exchanges were measured breath by breath using an online gas analysis system (MasterScreen CPX; CareFusion, Höchberg, Germany). Peak oxygen consumption (V̇O_2_peak) was calculated as the average of the three highest oxygen consumption values measured in 10-s averages. Criteria used for validation of V̇O_2_peak were completion of at least the first 8 min of the test protocol and a respiratory exchange ratio (carbon dioxide production/oxygen consumption) ≥ 1.1 [32]. Participants were instructed to maintain a cadence of 70–90 rpm and were verbally encouraged throughout the test.

#### Muscle Strength Test

Muscle strength was measured as the highest knee extensor peak torque (peak force relative to lever arm) measurement from five maximal isometric contractions (5-s duration, 60-s rest) using a dynamometer chair (Good Strength Metitur dynamometer chair; Metitur Oy, Jyväskylä, Finland). The test was performed on the dominant leg, with the knee fixed in a 60° flexion angle. Participants were instructed to slowly increase the force output and reach maximal effort after ~ 3 s. The lever arm was measured as the length from the lateral epicondyle to the center of the leg-supporting chalice.

### Endpoints

#### Key Secondary Endpoints

The key secondary endpoints in this paper were selected to represent changes in three inter-related constructs of physical fitness from randomization (week 0) to week 52:

(1) physical functional performance, time to complete the stair climb test (seconds); (2) cardiorespiratory fitness, VO_2peak_ relative to FFM (mL/min/kg FFM); and (3) muscle strength, knee extensor peak torque (Nm). Table S1 of the ESM outlines the conceptual framework, defining the overall term physical fitness as the ability to carry out daily tasks and perform physical activities in a highly functional state. Table S1 of the ESM also describes the specific constructs assessed and the rationale for the choice of endpoints.

#### Supportive Secondary Endpoints

Supportive secondary physical fitness endpoints included: self-reported physical functioning (Short Form 36 Health Survey); VO_2peak_ in absolute values; VO_2peak_ normalized to body weight (reported previously [29]); peak power output during the cycle test (exercise tolerance); and knee extensor peak torque normalized to body weight and to leg FFM. Endpoints from DXA were absolute appendicular FFM and appendicular FFM normalized to body weight or height. Details about endpoints are provided in the statistical analysis plan in the ESM.

#### Harms

Details on how harms were defined and assessed, and a summary of all harms, are provided in the trial protocol and primary trial report [29].

### Sample Size

The sample size was calculated based on the primary endpoint, a change in body weight [29]. For this secondary report, we estimated that the study would have at least 80% power to detect differences between groups in physical functional performance (0.8 s in the stair climb test), cardiorespiratory fitness (3.0 mL/min/kg FFM), and muscle strength (15 Nm). These differences have been associated with reduced all-cause and cardiovascular mortality and prevention of age-related declines [2, 33, 34] and were considered realistic to detect [7, 26], and they were therefore chosen as the minimum clinically relevant differences (see the statistical analysis plan in the ESM for details).

### Statistical Analysis

The primary analyses included all randomized participants who received at least one randomized treatment dose. All outcomes were analyzed using constrained linear mixed models with inherent pre-randomization adjustment to account for differences during the initial low-calorie diet. The model had time, a time-treatment interaction, and randomization factors as fixed effects, repeated effect for visit, and an unstructured covariance pattern. Missing data were assumed to be missing at random and handled implicitly by maximum likelihood estimation in the mixed model. Estimated mean differences between groups for the three key secondary endpoints were null-hypothesis tested and reported with *p*-values and a 95% confidence interval (CI). The Benjamini–Hochberg procedure [35] was used to control the false discovery rate (5%). Statistical significance was set at *α* = 0.05 (two-sided). Supportive secondary endpoints were not adjusted for multiple testing. Per-protocol supplementary analyses, excluding all protocol deviators, were performed for the key secondary endpoints. Per protocol was defined for exercise as at least 75% of 150 min/week at moderate intensity, 75 min/week at vigorous intensity, or an equivalent combination, and for medication as 2.4–3.0 mg for at least 75% of the intervention period. Multiple linear regression analyses were conducted to assess dose–response relationships between exercise volume (min/week of moderate-to-vigorous intensity exercise) and changes in body composition and physical fitness outcomes. Full details on statistical analysis methods are provided in the statistical analysis plan in the ESM.

## Results

### Trial Population

Between August 2016 and September 2018, 193 participants were randomized and received at least one dose of the randomized treatment (Fig. S1 of the ESM). Hereof, 28 participants (14.5%) were lost to the follow-up. Participant characteristics before and after the initial low-calorie diet are presented in Table 1.

**Table 1:** Participant characteristics before (week − 8) and after (week 0) a LCD

	Week − 8 [start of LCD] (*n* = 193)	Change during LCD [week − 8 to 0]	Week 0 (randomization)			
			Placebo (*n* = 48)	Exercise (*n* = 48)	Liraglutide (*n* = 49)	Exercise and liraglutide (*n* = 48)
Sex (male/female), *n* (%)	70/123 (36/64)		18/30 (38/62)	17/31 (35/65)	18/31 (37/63)	17/31 (35/65)
Age	42.9 (11.9)		42.8 (11.6)	43.0 (12.4)	43.2 (11.6)	42.3 (12.4)
Body composition						
Body mass index, kg/m^2^	37.0 (2.9)	− 4.3 (− 4.5, − 4.2)	32.3 (3.0)	32.7 (3.0)	32.7 (3.1)	32.8 (2.5)
Body weight, kg	109.7 (14.8)	− 13.0 (− 13.7, − 12.4)	96.9 (12.7)	96.8 (13.2)	95.1 (12.8)	98.1 (11.5)
Appendicular FFM, kg	28.6 (6.3)	− 2.07 (− 2.29, − 1.85)	27.2 (6.1)	27.0 (5.2)	25.9 (5.5)	26.6 (5.7)
Appendicular FFM, kg/kg BW*100	26.0 (3.2)	1.42 (1.26, 1.58)	27.7 (3.9)	27.9 (3.4)	27.1 (3.4)	27.0(3.6)
Appendicular FFM, kg/m^2^	9.6 (1.3)	− 0.68 (− 0.75, − 0.61)	8.9 (1.1)	9.1 (1.1)	8.8 (1.1)	8.8 (1.0)
Physical functional performance						
Stair climb test performance, s	15.9 (3.4)	− 0.89 (− 1.09, − 0.69)	14.9 (3.0)	14.8 (3.9)	15.0 (3.6)	15.3 (2.5)
Physical functioning score^a^	85.6 (12.5)	5.0 (3.8, 6.2)	95 (90; 100)	95 (85;100)	90 (85;100)	90 (85; 95)
Cardiorespiratory fitness						
V̇O_2peak_, mL/min	2543 (589)	− 115 (− 155, − 74)	2479 (662)	2551 (705)	2372 (537)	2363 (531)
V̇O_2peak_, mL/min/kg FFM	39.0 (5.5)	1.4 (0.7, 2.0)	40.4 (5.7)	41.8 (7.7)	40.1 (6.3)	38.9 (6.3)
V̇O_2peak_, mL/min/kg BW	23.2 (4.1)	2.0 (1.6, 2.4)	25.5 (5.1)	26.6 (6.0)	25 (4.6)	23.9 (4.3)
Peak watt	199 (55)	− 5.6 (− 7.9, − 3.4)	195 (62)	203 (61)	186 (56)	190 (45)
Muscle strength						
Isometric knee extensor peak torque, Nm	196.9 (64.7)	− 0.4 (− 3.5, 2.8)	194.9 (69.7)	209 (63.7)	185.3 (68.4)	197.2 (62.7)
Isometric knee extensor peak torque, Nm/kg leg FFM	17.7 (3.8)	1.27 (0.93, 1.62)	18.7 (4.3)	19.9 (4.4)	18.4 (5.0)	18.9 (3.7)
Isometric knee extensor peak torque, Nm/kg BW	1.8 (0.5)	0.24 (0.21, 0.28)	2.0 (0.6)	2.2 (0.6)	1.9 (0.6)	2 (0.5)

*BW* body weight, *CI* confidence interval, *FFM* fat-free mass, *LCD* low-calorie diet, *min* minute, *Nm* newton meter, *s* seconds, *V̇O*_*2peak*_ Peak oxygen consumption

Values at week − 8 and week 0 are observed mean (standard deviation), median (first quartile; third quartile), or count (%) in the full analysis set

Changes during LCD (from week − 8 to 0) are estimated mean (95% CI) derived from constrained linear mixed models with inherent pre-randomization adjustment and the following fixed effects: time, time-treatment interaction, sex, and age group

^a^Physical function score from the Short Form 36 Health Survey, range from 0 to 100, with higher scores indicating better physical functioning

### Adherence to Interventions

**Fig. 1:**
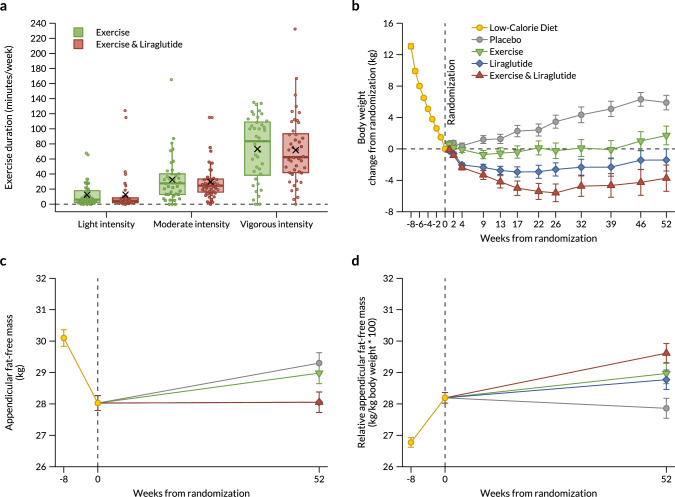
Exercise duration and body composition. **a** Box plots of exercise duration after the program was fully implemented (weeks 7–52 after randomization). Data are shown for participants who attended week 52 (*n* = 40 for exercise alone and *n* = 44 for combined exercise and liraglutide). Intensities were defined as the percentage of maximum heart rate derived from an incremental maximal cycle test: light-intensity exercise, 50–63%; moderate-intensity exercise, 64–76%; and vigorous-intensity exercise, ≥ 77%. In the box plots, the *lines inside the boxes* indicate medians; the *boxes* are interquartile ranges (from the first quartile to the third quartile); the *crosses* are observed means; and the *whiskers* are the smallest and largest values within 1.5 × the interquartile range. *Dots* indicate individual participant values. **b** Observed mean ± standard error changes relative to randomization in body weight during the low-calorie diet (week − 8 to 0) for all participants and from randomization (week 0) to week 52 for the four randomized groups. **c** Least-squares mean ± standard error appendicular fat-free mass over time. **d** Least-squares mean ± standard error appendicular fat-free mass relative to body weight over time. Data in **b–d** are from the full analysis set (all randomized irrespective of completion and treatment discontinuation), and data in **c** and **d** were derived from constrained linear mixed models (see Sect. 2.8 for details)

After the low-calorie diet, the structured exercise program started with a gradual increase in exercise volume over the first 6 weeks after randomization. From weeks 7 to 52, the participants randomized to an exercise group (exercise alone or combined with liraglutide) performed a median 2.65 session/week, of which a median (first quartile; third quartile) 108 min/week (59; 141) was at moderate-to-vigorous intensity exercise, with no significant difference between groups (Fig. 1A). Hereof, the majority (median 71 min/week) was of vigorous intensity. The median light-intensity exercise performed was 5 min/week (2; 11). Of the 40 and 45 participants who were randomized to exercise alone or combined treatment and completed the study, 26 (65%) and 29 (64%) were per-protocol.

A mean liraglutide dose between 2.4 mg and 3.0 mg once daily was reached by 35 participants (85%) with liraglutide alone and by 40 participants (89%) with the combined treatment. One participant in the liraglutide-alone group and two participants in the combined treatment group discontinued the medication.

### Body Composition

Changes in body weight have been reported previously [29] and are presented descriptively in Fig. 1B. The 8-week low-calorie diet resulted in a 13.1-kg weight loss. From randomization to week 52, the observed changes in body weight were 5.9 kg with placebo, 1.7 kg with exercise alone, − 1.4 kg with liraglutide alone, and − 3.7 kg with the combined treatment. Appendicular FFM increased with placebo and with exercise alone (Fig. 1C). Appendicular FFM relative to body weight increased in the three active treatment groups compared with placebo and with greater increases during the combined treatment than with liraglutide alone (Fig. 1D).

### Physical Fitness Endpoints

#### Physical Functional Performance

**Fig. 2:**
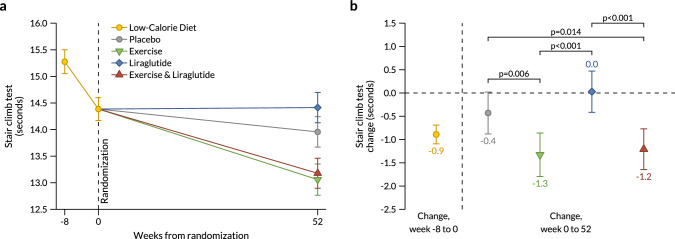
Physical functional performance. **a** Physical functional performance over time measured as the time to complete a stair climb test. Values are least square mean ± standard error. **b** Least square mean ± 95% confidence interval changes in the stair climb test in response to the low-calorie diet for all participants (weeks − 8 to 0) and from randomization (week 0) to week 52 for the four randomized treatment groups. *P*-values are provided for statistically significant between-group differences (*P* < 0.05, and the Benjamini–Hochberg procedure to control the false discovery rate). All data were derived from constrained linear mixed models (see Sect. 2.8 for details), which included all randomized participants who received at least one randomized treatment dose, irrespective of completion and treatment adherence

Physical functional performance, as measured by the stair climb test, improved following the low-calorie diet (Table 1 and Fig. 2). Fifty-two weeks after randomization, combined exercise and liraglutide treatment decreased the time to complete the stair climb test compared with liraglutide alone (− 1.24 s; 95% CI − 1.85, − 0.62) and compared with placebo (− 0.78 s; 95% CI − 1.39, − 0.16) (Fig. 2A, B). Similarly, exercise alone reduced the time to complete the stair climb test compared with liraglutide alone (− 1.36 s; 95% CI − 1.99, − 0.72) and compared with placebo (− 0.90 s; 95% CI − 1.53, − 0.26). Liraglutide alone did not improve the stair climb test performance compared with placebo (+ 0.46 s; 95% CI − 0.16, 1.08). The per-protocol analyses consistently supported the results of the primary analyses and showed larger effect sizes with exercise alone and combined with liraglutide (Table S2 of the ESM).

The combined treatment improved self-reported physical functioning compared with liraglutide alone and placebo (Table 2 and Table S3 of the ESM). Liraglutide alone did not improve self-reported physical functioning.

**Table 2:** Key secondary and supportive secondary outcomes

	Placebo	Exercise	Liraglutide	Exercise and liraglutide
**Key secondary outcomes**				
*Physical functional performance (stair climb test performance), s*				
Within-group change, weeks 0–52	− 0.43 (− 0.88, 0.02)	− 1.33 (− 1.79, − 0.86)	0.03 (− 0.42, 0.47)	− 1.21 (− 1.65, − 0.77)
ETD vs placebo	NA	− 0.90 (− 1.53, − 0.26) *p* = 0.006^a^	0.46 (− 0.16, 1.08) *p* = 0.147	− 0.78 (− 1.39, − 0.16) *p* = 0.014^a^
ETD vs exercise	NA	NA	1.36 (0.72, 1.99) *p* < 0.001^a^	0.12 (− 0.51, 0.75) *p* = 0.71
ETD vs liraglutide	NA	NA	NA	− 1.24 (− 1.85, − 0.62) *p* < 0.001^a^
*Cardiorespiratory fitness (V̇O*_*2peak*_* normalized to FFM), mL/min/kg FFM*				
Within-group change, weeks 0–52	− 1.1 (− 3.0, 0.9)	5.0 (3.2, 6.9)	1.2 (− 0.7, 3.1)	4.2 (2.4, 5.9)
ETD vs placebo	NA	6.1 (3.5, 8.7) *p* < 0.001^a^	2.3 (− 0.4, 4.9) *p* = 0.089	5.3 (2.7, 7.8) *p* = 0.001^a^
ETD vs exercise	NA	NA	− 3.8 (− 6.4, − 1.2) *p* = 0.004^a^	− 0.8 (− 3.3, 1.7) *p* = 0.51
ETD vs liraglutide	NA	NA	NA	3.0 (0.5, 5.5) *p* = 0.019^a^
*Muscle strength (isometric knee extensor peak torque), Nm*				
Within-group change, weeks 0–52	− 4.2 (− 11.2, 2.8)	1.2 (− 6.0, 8.4)	− 2.3 (− 9.1, 4.6)	− 4.5 (− 11.3, 2.3)
ETD vs placebo	NA	5.4 (− 4.5, 15.3) *p* = 0.28	1.9 (− 7.8, 11.6) *p* = 0.70	− 0.3 (− 9.9, 9.3) *p* = 0.95
ETD vs exercise	NA	NA	− 3.5 (− 13.3, 6.3) *p* = 0.48	− 5.7 (− 15.5, 4.0) *p* = 0.25
ETD vs liraglutide	NA	NA	NA	− 2.2 (− 11.7, 7.3) *p* = 0.64
**Supportive secondary outcomes (within-group changes from randomization [week 0] to week 52)**				
Physical functioning score^d^	− 0.6 (− 3.1, 1.9)	2.0 (− 0.5, 4.6)	0.0 (− 2.4, 2.4)	3.5 (1.2, 5.8)^b,c^
V̇O_2peak_, mL/min/kg BW	− 0.9 (− 2.4, 0.7)	3.9 (2.5, 5.4)^b,c^	1.3 (− 0.2, 2.8)	4.5 (3.1, 5.9)^b,c^
V̇O_2peak_, mL/min	72 (− 49, 194)	378 (262, 493)^b,c^	73 (− 43, 188)	290 (181, 399)^b,c^
Peak watt	6.2 (− 2.7, 15.0)	33.4 (24.4, 42.3)^b,c^	2.2 (− 6.5, 11.0)	28.1 (19.8, 36.4)^b,c^
Peak torque, Nm/kg leg FFM	− 1.37 (− 2.08, − 0.66)	− 0.53 (− 1.26, 0.19)	− 0.30 (− 0.99, 0.40)^b^	− 0.50 (− 1.19, 0.18)
Peak torque, Nm/kg BW	− 0.17 (− 0.26, − 0.08)	− 0.01 (− 0.10, 0.08)^b^	0.02 (− 0.07, 0.11)^b^	0.07 (− 0.02, 0.16)^b^
Appendicular FFM, kg	1.27 (0.80, 1.74)	0.95 (0.45, 1.44)^c^	0.03 (− 0.43, 0.48)^b^	0.03 (− 0.42, 0.48)^b^
Appendicular FFM, kg/kg BW*100	− 0.33 (− 0.89, 0.22)	0.78 (0.21, 1.34)^b^	0.58 (0.04, 1.12)^b^	1.42 (0.89, 1.95)^b,c^
Appendicular FFM, kg/m^2^	0.42 (0.26, 0.58)	0.31 (0.15, 0.47)^c^	− 0.01 (− 0.17, 0.14)^b^	− 0.01 (− 0.16, 0.14)^b^

*BW* body weight, *CI* confidence interval, *ETD* estimated treatment difference, *FFM* fat-free mass, *min* minute, *NA* not applicable, *Nm* newton meter, *s* seconds, *V̇O2*_*peak*_ peak oxygen consumption

Values are mean (95% CI) changes from randomization (week 0) to week 52 in the full analysis set estimated from constrained linear mixed models with inherent pre-randomization adjustment and the following fixed effects: time, time–treatment interaction, sex, and age group

For the three key secondary outcomes, ETDs with 95% CI are reported as between-group differences with corresponding *p*-values controlled for the false discovery rate using the Benjamini–Hochberg method

Between-group mean differences (95% CI) for all supportive outcomes are available in Table S3 of the ESM

^a^Statistically significant at the 5% false discovery rate using the Benjamini–Hochberg procedure

^b^The 95% CI of the comparison to placebo for a supportive outcome does not include 0, indicating a statistically significant difference

^c^The 95% CI of the comparison to liraglutide for a supportive outcome does not include 0, indicating a statistically significant difference

^d^Physical function score from the Short Form 36 Health Survey, range from 0 to 100, with higher scores indicating better physical functioning

#### Cardiorespiratory Fitness

**Fig. 3:**
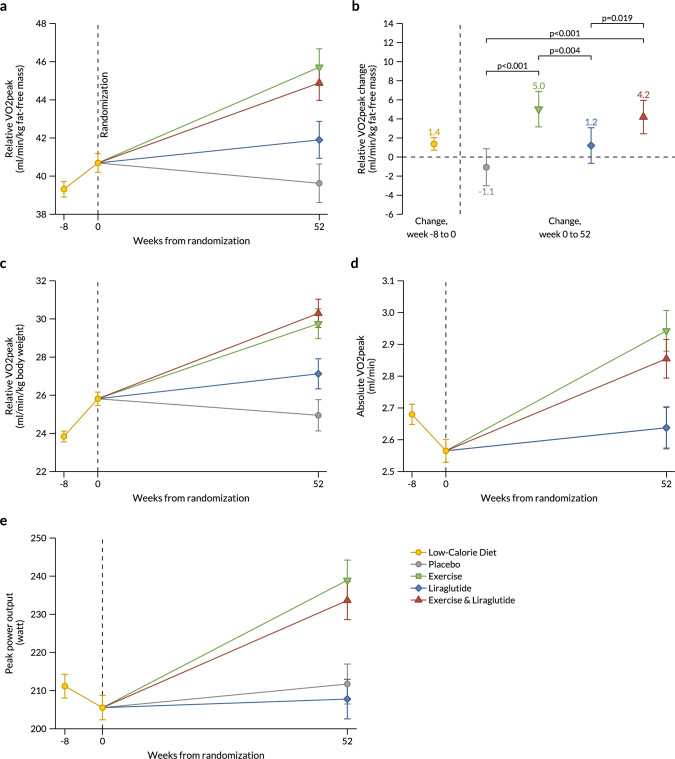
Cardiorespiratory fitness. **a** Cardiorespiratory fitness over time expressed as least square mean ± standard error peak oxygen consumption (V̇O_2peak_) relative to fat-free mass. **b** Least square mean ± 95% confidence interval changes in V̇O _2peak_ relative to fat-free mass in response to the low-calorie diet for all participants (week − 8 to 0) and from randomization (week 0) to week 52 for the four randomized treatment groups. *P*-values are provided for statistically significant between-group differences (*P* < 0.05, and the Benjamini–Hochberg procedure to control the false discovery rate). **c** Least square mean ± standard error V̇O_2peak_ relative to body weight over time. **d** Least square mean ± standard error absolute V̇O_2peak_ over time. **e** Exercise tolerance expressed as Least square mean ± standard error peak power output reached during the incremental cycle test over time. All data were derived from constrained linear mixed models (see Sect. 2.8 for details), which included all randomized participants who received at least one randomized treatment dose, irrespective of completion and treatment adherence. *min* minutes

Fifty-two weeks after randomization, the combined exercise and liraglutide treatment increased cardiorespiratory fitness, measured as V̇O2_peak_ relative to FFM, by 3.0 mL/min/kg FFM (95% CI 0.5, 5.5) compared with liraglutide alone and by 5.3 mL/min/kg FFM (95% CI 2.7, 7.8) compared with placebo (Fig. 3A, B and Table 2). Exercise alone led to similar improvements in cardiorespiratory fitness as the combined treatment, superior to liraglutide alone (3.8 mL/min/kg FFM [95% CI 1.2, 6.4]) and placebo (6.1 mL/min/kg FFM [95% CI 3.5, 8.7]). Liraglutide alone did not significantly improve cardiorespiratory fitness compared to placebo (2.3 mL/min/kg FFM [95% CI − 0.4, 4.9]). The combined treatment and exercise alone increased V̇O_2peak_ relative to body weight (Fig. 3C), absolute V̇O_2peak_ (Fig. 3D), and exercise tolerance (Fig. 3E) as compared with placebo and liraglutide alone. Liraglutide alone did not improve any of the endpoints related to cardiorespiratory fitness. The supplementary per-protocol analyses, excluding protocol deviators, consistently supported the results of the primary analyses and showed larger estimated improvements with the combined treatment and exercise alone (Table S2 of the ESM).

#### Muscle Strength

**Fig. 4:**
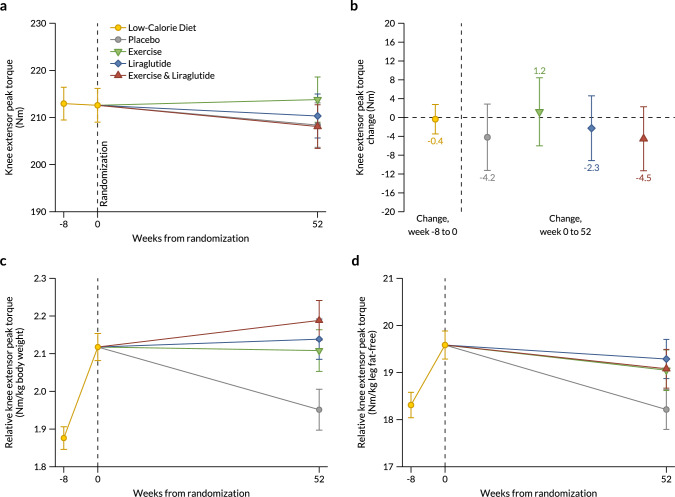
Muscle strength. **a** Absolute maximal muscle strength over time expressed as least square mean ± standard error knee extensor peak torque. **b** Least square mean ± 95% confidence interval changes in knee extensor peak torque in response to the low-calorie diet for all participants (week − 8 to 0) and from randomization (week 0) to week 52 for the four randomized treatment groups. No between-group comparisons were statistically significant. **c** Least square mean ± standard error knee extensor peak torque relative to body weight over time. **d** Muscle quality expressed as least square mean ± standard error knee extensor peak torque relative to leg fat-free mass over time. All data were derived from constrained linear mixed models (see Sect. 2.8 for details), which included all randomized participants who received at least one randomized treatment dose, irrespective of completion and treatment adherence. *Nm* newton meter

Muscle strength, as measured by the isometric knee extensor peak torque, did not change significantly in any of the groups (Fig. 4A, B). Muscle strength relative to body weight increased with the three active treatments compared with placebo (Fig. 4C). Muscle quality decreased with placebo but was unchanged with the active treatments (Fig. 4D).

#### Dose–Response Effects of Exercise on Physical Fitness and Body Composition

The amount of moderate-to-vigorous intensity exercise performed in the study was positively associated with improved physical functional performance and cardiorespiratory fitness (Table 3). Specifically, each 10-min/week increment of moderate-to-vigorous intensity exercise was associated with a 0.33-mL/min/kg FFM increase in cardiorespiratory fitness and a 0.057-s decrease in time to complete the stair climb test. In addition, increased exercise volume was associated with greater reductions in body weight, fat mass, and fat percentage, but increased appendicular FFM relative to body weight.

**Table 3:** Associations between moderate-to-vigorous intensity exercise amount and changes in fitness outcomes and body composition from randomization to week 52

Explanatory variable: moderate-to-vigorous intensity exercise (10 × min/week)		
Dependent variable (change from randomization to week 52)	Unstandardized *β* (95% CI)	Standardized *β* (95% CI)
V̇O2_peak_, mL/min/kg FFM	0.33 (0.10, 0.56)^a^	0.29 (0.09, 0.49)^a^
V̇O2_peak_, mL/min	18.2 (3.2, 33.2)^a^	0.26 (0.04, 0.47)^a^
Stair climb test performance (s)	− 0.057 (− 0.097, − 0.017)^a^	− 0.24 (− 0.41, − 0.07)^a^
Knee extensor strength (Nm)	− 0.56 (− 1.50, 0.38)	− 0.14 (− 0.37, 0.09)
Body weight (kg)	− 0.38 (− 0.71, − 0.05)^a^	− 0.24 (− 0.45, − 0.03)^a^
Fat percentage (% points)	− 0.18 (− 0.32, − 0.04)^a^	− 0.30 (− 0.53, − 0.06)^a^
Fat mass (kg)	− 0.28 (− 0.53, − 0.03)^a^	− 0.26 (− 0.49, − 0.03)^a^
FFM (kg)	− 0.05 (− 0.19, 0.10)	− 0.07 (− 0.30, 0.15)
Appendicular FFM (kg)	0.01 (− 0.05, 0.08)	0.05 (− 0.18, 0.27)
Appendicular FFM (kg/kg BW*100)	0.13 (0.06, 0.20)^a^	0.40 (0.19, 0.62)^a^

*BW* body weight, *CI* confidence interval, *FFM* fat-free mass, *min* minute, *Nm* newton meter, *s* seconds, *V̇O*_*2peak*_ peak oxygen consumption

Moderate-to-vigorous intensity was defined as at least 64% of maximum heart rate

Data are presented as unstandardized and standardized regression coefficients [*β*] (95% CI) for all participants randomized to exercise (exercise and placebo or exercise and liraglutide)

The unstandardized *β* indicates the associated change in the dependent variable (in the original unit of measurement) for each 10-min increment in moderate-to-vigorous intensity exercise per week

The standardized *β* indicates the associated change (in standard deviations) in the dependent variable for each standard deviation increment in moderate-to-vigorous intensity exercise per week

Regression coefficients were estimated from a multiple regression analysis with moderate-to-vigorous-intensity physical activity (min/week), age, sex, liraglutide treatment (yes/no), and randomization value of the dependent variable as covariates

^a^The 95% CI does not include 0, indicating a statistically significant association

## Discussion

In this weight maintenance trial, combined exercise and liraglutide treatment improved physical fitness in terms of physical functional performance and cardiorespiratory fitness as compared with liraglutide alone. Similar improvements were observed with exercise alone, whereas liraglutide alone had no effect on these physical fitness endpoints. Muscle strength relative to body weight increased across all active treatment groups compared with placebo, because of decreased body weight and preserved absolute strength. These findings underscore the value of structured exercise in enhancing physical fitness during obesity pharmacotherapy and that, despite significant weight loss, pharmacotherapy alone was insufficient to improve physical fitness beyond what was initially achieved through diet-induced weight loss.

### Physical Functional Performance

The structured exercise program added to liraglutide reduced the time to complete a stair climb test by 8.6%. The stair climb test was a performance-based measure to assess physical functional performance; in addition to physical functioning, the test reflects anaerobic power, lower limb strength, balance, agility, and mobility [36]. Exercise and liraglutide as separate treatments maintained weight loss compared with regain in the placebo group, and the combined treatment showed the largest weight reduction compared with placebo. However, we observed similar improvements with combined exercise and liraglutide treatment as with exercise alone, but liraglutide treatment alone had no effect. Thus, the improved performance with the combined treatment seemed driven by exercise independent of weight loss. Although we did not directly assess weight-independent adaptations, the observed improvements with exercise irrespective of liraglutide treatment could be related to improved mobility, balance, and lower-limb power as a result of neuromuscular and metabolic adaptations to exercise [37]. The impaired physical functioning and mobility often experienced by people with obesity reduce health-related quality of life [5]. Stair climb tests are generally sensitive to detect declines in physical functioning and mobility limitations in mid/late life, which are accelerated by obesity [1, 2, 38, 39]. Stair climb performance declines up to 1% per year in mid and late life [2, 38], indicating that the observed improvements with exercise may potentially translate into a clinically meaningful prevention of age-related decline in physical functioning. The magnitude of improvement aligns with the 5–10% improvements in physical functioning typically observed in response to exercise interventions alone or as an add-on to calorie restriction [26, 40–42].

Exercise combined with liraglutide also improved self-reported physical functioning compared with liraglutide alone. The physical functioning score relates to health limitations to performing vigorous and moderate activities, walking shorter distances, lifting groceries, getting dressed, bending/kneeling, and climbing stairs. The phase III trials of liraglutide, semaglutide, and tirzepatide all showed improvements in self-reported physical functioning [14, 43, 44]. These three incretin-based medications also previously improved 6-min walking distance in patients with overweight or obesity and heart failure [45–47]. Our study differs from these studies because the study participants were younger without heart disease, and therefore had higher baseline physical functioning. In addition, participants in our study improved their physical function during the initial weight loss. Liraglutide maintained but did not substantially extend this weight loss, which could have limited the detection of any further functional benefits. Despite producing a 6.8-kg weight difference relative to placebo, liraglutide did not confer additional improvements in physical function. In the SCALE IBT trial, the addition of liraglutide to intensive behavioral therapy did not improve walking distance [15]. This study had a study population similar to that of the present study, collectively suggesting that, for obesity care, liraglutide alone is not effective for improving physical functioning.

### Cardiorespiratory Fitness

The combined exercise and liraglutide treatment also significantly improved cardiorespiratory fitness by 4.2 mL/min/kg FFM, corresponding to a 10.3% within-group improvement, whereas liraglutide alone had no significant effect. The magnitude of improvement in cardiorespiratory fitness is clinically important. Indeed, V̇O2_peak_ is directly related to FFM, and normalizing to FFM reflects fitness related to exercise performance and cardiorespiratory function and has high prognostic value for mortality [33, 48–50]. In participants followed for a mean of 19 years, all-cause and cardiovascular disease mortality were 16% and 8% lower, respectively, per 1 mL/min/kg FFM higher cardiorespiratory fitness [33]. Exercise improved cardiorespiratory fitness similarly with and without liraglutide treatment, indicating that the fitness-enhancing effects of exercise will also appear during obesity pharmacotherapy but that liraglutide treatment does not enhance these benefits further. Thus, exercise was necessary for improved cardiorespiratory fitness during obesity pharmacotherapy. As liraglutide alone did not improve cardiorespiratory fitness compared with placebo, we speculate that the improvements observed with combined exercise and liraglutide were mainly due to exercise-induced physiological adaptations [51], rather than weight loss per se. Exercise adherence was similar with and without liraglutide, suggesting that obesity pharmacotherapy did not interfere with the ability to engage in vigorous-intensity exercise.

### Muscle Strength and FFM

During the weight maintenance phase, appendicular FFM was unchanged with the combined treatment despite an additional weight loss. Appendicular FFM normalized to body weight, a surrogate marker of muscle mass used to assess sarcopenia risk [52], increased in all active groups, but more with combined exercise and liraglutide treatment than with liraglutide alone. Absolute muscle strength was unchanged throughout the study, and body weight was decreased in the active treatment groups compared with placebo. As a result, strength relative to body weight increased in the three active treatment groups compared with placebo. Our data suggest that weight maintenance with liraglutide alone or combined with exercise preserved muscle function. The muscle-strengthening exercises in the present program were performed as circuit training and not based on repetition maximum zones. A larger component of progressive heavy-resistance exercise with intensities based on repetition maximum would likely have been more effective for muscle hypertrophy and strength gain [53].

### Exercise Volume

A median of 108 min/week of moderate-to-vigorous intensity exercise improved physical fitness and maintained weight loss. This volume is lower than the 200–300 min/week of physical activity generally recommended to maintain weight loss [54–56]. A dose–response association was observed between exercise performed and improvements in both physical fitness and body composition outcomes. Thus, more exercise led to larger improvements in physical fitness and better maintenance of healthy weight loss. However, even lower exercise volumes, especially going from no to little vigorous-intensity exercise, may be meaningful for physical fitness and body composition in relation to obesity care.

### Weight Loss and Physical Fitness

In response to the initial 13.1 kg diet-induced weight loss, absolute V̇O_2peak_ and exercise tolerance were slightly decreased. Peak oxygen consumption relative to body weight or FFM improved together with physical functional performance. Overall, the weight loss led to some improvements and some impairments in physical fitness. Although there seemed to be more benefits, a large calorie restriction without increased physical activity was not an optimal strategy for physical fitness.

### Strengths and Limitations

Strengths of this trial were the randomized controlled design with direct comparisons between separate and combined exercise and GLP-1 RA treatment, the structured and monitored exercise program, high adherence to the interventions and low dropout rates, and that we employed objective validated tests to measure physical function, muscle strength, and cardiorespiratory fitness.

The trial also has some limitations. As adults aged 18–65 years with no known serious chronic diseases were included, the findings may not be generalized to people above 65 years of age or populations with other co-existing comorbidities. Some physical fitness outcomes (stair climb performance and VO_2peak_ normalized to FFM) improved during the low-calorie diet, which may have reduced the potential for further improvement during the randomized period. The exercise intervention may not have been optimal for maximizing muscle strength or hypertrophy. We did not directly assess physiological adaptations, and any discussion of mechanisms underlying the observed changes in physical fitness is speculative. Finally, although physical fitness was assessed using validated high-quality measures, additional functional tests such as the short physical performance battery, timed up-and-go, and handgrip strength could have provided further insights into functional domains and upper-body strength.

### Clinical Implications

As incretin-based pharmacotherapy becomes increasingly widespread in obesity care, understanding how to optimize patient outcomes is essential. Using high-quality assessments of physical fitness, our trial provides evidence that pharmacotherapy requires structured exercise integration owing to a clinically meaningful improvement in physical fitness. Such a structured approach should be delivered by qualified exercise professionals and provide tailored support, access to facilities, and consider challenges related to excess adiposity rather than relying on general physical activity recommendations.

## Conclusions

One year of weight maintenance with exercise and GLP-1RA combined significantly improved physical functional performance and cardiorespiratory fitness. The benefits were driven by exercise, and GLP-1RA alone did not improve these physical fitness constructs. Absolute muscle strength was preserved after GLP-1RA treatments alone and combined with exercise despite weight reduction.

## Supplementary Information

Below is the link to the electronic supplementary material.

Supplementary file1 (PDF 812 KB)

Supplementary file2 (PDF 822 KB)

## Declarations

### Availability of Data and Material

Data from the study are not available for download because of restrictions under the General Data Protection Regulation. De-identified data, under the General Data Protection Regulation and local regulations of Denmark and the University of Copenhagen, may be available for research collaboration purposes upon reasonable request to the corresponding author, and will require the approval of the corresponding author and the completion of a data processing agreement.

## References

[1] Pataky Z, Armand S, Müller-Pinget S, Golay A, Allet L. Effects of obesity on functional capacity. Obesity Silver Spring. 2014;22(1):56–62. 23794214 10.1002/oby.20514

[2] Lange-Maia BS, Karvonen-Gutierrez CA, Strotmeyer ES, et al. Factors influencing longitudinal stair climb performance from midlife to early late life: the study of women’s health across the nation Chicago and Michigan sites. J Nutr Health Aging. 2019;23(9):821–8. 31641731 10.1007/s12603-019-1254-2 PMC6818752

[3] Wei M, Kampert JB, Barlow CE, et al. Relationship between low cardiorespiratory fitness and mortality in normal-weight, overweight, and obese men. JAMA. 1999;282(16):1547–53. 10546694 10.1001/jama.282.16.1547

[4] Sloan RA, Sawada SS, Martin CK, Church T, Blair SN. Associations between cardiorespiratory fitness and health-related quality of life. Health Qual Life Outcomes. 2009;7(1):47. 19476640 10.1186/1477-7525-7-47 PMC2695434

[5] Forhan M, Gill SV. Obesity, functional mobility and quality of life. Best Pract Res Clin Endocrinol Metab. 2013;27(2):129–37. 23731875 10.1016/j.beem.2013.01.003

[6] Vaughan L, Leng X, La Monte MJ, Tindle HA, Cochrane BB, Shumaker SA. Functional independence in late-life: maintaining physical functioning in older adulthood predicts daily life function after age 80. J Gerontol A Biol Sci Med Sci. 2016;71(Suppl. 1):S79-86. 26858328 10.1093/gerona/glv061 PMC5865534

[7] Ross R, Blair SN, Arena R, et al. Importance of assessing cardiorespiratory fitness in clinical practice: a case for fitness as a clinical vital sign: a scientific statement from the American Heart Association. Circulation. 2016;134(24):e653–99. 27881567 10.1161/CIR.0000000000000461

[8] García-Hermoso A, Cavero-Redondo I, Ramírez-Vélez R, et al. Muscular strength as a predictor of all-cause mortality in an apparently healthy population: a systematic review and meta-analysis of data from approximately 2 million men and women. Arch Phys Med Rehabil. 2018;99(10):2100-13.e5. 29425700 10.1016/j.apmr.2018.01.008

[9] Molari M, Fernandes KBP, Marquez AdS, Probst VS, Bignardi PR, Teixeira DdC. Impact of physical and functional fitness on mortality from all causes of physically independent older adults. Arch Gerontol Geriatr. 2021;97:104524. 34547537 10.1016/j.archger.2021.104524

[10] Weeldreyer NR, De Guzman JC, Paterson C, Allen JD, Gaesser GA, Angadi SS. Cardiorespiratory fitness, body mass index and mortality: a systematic review and meta-analysis. Br J Sports Med. 2025;59(5):339–46. 39537313 10.1136/bjsports-2024-108748 PMC11874340

[11] Oktay AA, Lavie CJ, Kokkinos PF, Parto P, Pandey A, Ventura HO. The interaction of cardiorespiratory fitness with obesity and the obesity paradox in cardiovascular disease. Prog Cardiovasc Dis. 2017;60(1):30–44. 28502849 10.1016/j.pcad.2017.05.005

[12] Busetto L, Dicker D, Frühbeck G, et al. A new framework for the diagnosis, staging and management of obesity in adults. Nat Med. 2024;30(9):2395–9. 38969880 10.1038/s41591-024-03095-3

[13] Jensen SBK, Janus C, Lundgren JR, et al. Exploratory analysis of eating- and physical activity-related outcomes from a randomized controlled trial for weight loss maintenance with exercise and liraglutide single or combination treatment. Nat Commun. 2022;13(1):4770. 35970829 10.1038/s41467-022-32307-y PMC9378667

[14] Pi-Sunyer X, Astrup A, Fujioka K, et al. A randomized, controlled trial of 3.0 mg of liraglutide in weight management. N Engl J Med. 2015;373(1):11–22. 26132939 10.1056/NEJMoa1411892

[15] Wadden TA, Tronieri JS, Sugimoto D, et al. Liraglutide 3.0 mg and intensive behavioral therapy (IBT) for obesity in primary care: the SCALE IBT randomized controlled trial. Obesity. 2020;28(3):529–36. 32090517 10.1002/oby.22726 PMC7065111

[16] Wadden TA, Hollander P, Klein S, et al. Weight maintenance and additional weight loss with liraglutide after low-calorie-diet-induced weight loss: the SCALE maintenance randomized study. Int J Obes (Lond). 2013;37(11):1443–51. 23812094 10.1038/ijo.2013.120

[17] Müllertz ALO, Sandsdal RM, Jensen SBK, Torekov SS. Potent incretin-based therapy for obesity: a systematic review and meta-analysis of the efficacy of semaglutide and tirzepatide on body weight and waist circumference, and safety. Obes Rev. 2024;25(5):e13717. 38463003 10.1111/obr.13717

[18] Dubin RL, Heymsfield SB, Ravussin E, Greenway FL. Glucagon-like peptide-1 receptor agonist-based agents and weight loss composition: filling the gaps. Diabetes Obes Metab. 2024;26(12):5503–18. 39344838 10.1111/dom.15913

[19] Mechanick JI, Butsch WS, Christensen SM, et al. Strategies for minimizing muscle loss during use of incretin-mimetic drugs for treatment of obesity. Obes Rev. 2024;26(1):e13841. 39295512 10.1111/obr.13841 PMC11611443

[20] Hope DCD, Tan TM. Skeletal muscle loss and sarcopenia in obesity pharmacotherapy. Nat Rev Endocrinol. 2024;20(12):695–6. 39300316 10.1038/s41574-024-01041-4

[21] Tinsley GM, Heymsfield SB. Fundamental body composition principles provide context for fat-free and skeletal muscle loss with GLP-1 RA treatments. J Endocr Soc. 2024;8(11):bvae164. 39372917 10.1210/jendso/bvae164 PMC11450469

[22] Pandey A, Patel KV, Segar MW, et al. Effect of liraglutide on thigh muscle fat and muscle composition in adults with overweight or obesity: results from a randomized clinical trial. J Cachexia Sarcopenia Muscle. 2024;15(3):1072–83. 38561962 10.1002/jcsm.13445 PMC11154779

[23] Sattar N, Neeland IJ, Dahlqvist Leinhard O, et al. Tirzepatide and muscle composition changes in people with type 2 diabetes (SURPASS-3 MRI): a post-hoc analysis of a randomised, open-label, parallel-group, phase 3 trial. Lancet Diabetes Endocrinol. 2025;13(6):482–93. 40318682 10.1016/S2213-8587(25)00027-0

[24] Newman AB, Kupelian V, Visser M, et al. Strength, but not muscle mass, is associated with mortality in the Health, Aging and Body Composition Study cohort. J Gerontol A Biol Sci Med Sci. 2006;61(1):72–7. 16456196 10.1093/gerona/61.1.72

[25] Mesinovic J, Hurst C, Leung GKW, Ryan JR, Daly RM, Scott D. Exercise and dietary recommendations to preserve musculoskeletal health during weight loss in adults with obesity: a practical guide. Rev Endocr Metab Disord. 2025;26(5):785–803. 40434574 10.1007/s11154-025-09968-3 PMC12534310

[26] Villareal DT, Chode S, Parimi N, et al. Weight loss, exercise, or both and physical function in obese older adults. N Engl J Med. 2011;364(13):1218–29. 21449785 10.1056/NEJMoa1008234 PMC3114602

[27] Weiss EP, Jordan RC, Frese EM, Albert SG, Villareal DT. Effects of weight loss on lean mass, strength, bone, and aerobic capacity. Med Sci Sports Exerc. 2017;49(1):206–17. 27580151 10.1249/MSS.0000000000001074 PMC5161655

[28] O’Donoghue G, Blake C, Cunningham C, Lennon O, Perrotta C. What exercise prescription is optimal to improve body composition and cardiorespiratory fitness in adults living with obesity? A network meta-analysis. Obes Rev. 2021;22(2):e13137. 32896055 10.1111/obr.13137 PMC7900983

[29] Lundgren JR, Janus C, Jensen SBK, et al. Healthy weight loss maintenance with exercise, liraglutide, or both combined. N Engl J Med. 2021;384(18):1719–30. 33951361 10.1056/NEJMoa2028198

[30] Jensen SBK, Lundgren JR, Janus C, et al. Protocol for a randomised controlled trial of the combined effects of the GLP-1 receptor agonist liraglutide and exercise on maintenance of weight loss and health after a very low-calorie diet. BMJ Open. 2019;9(11):e031431. 31678947 10.1136/bmjopen-2019-031431 PMC6830609

[31] Bull FC, Al-Ansari SS, Biddle S, et al. World Health Organization 2020 guidelines on physical activity and sedentary behaviour. Br J Sports Med. 2020;54(24):1451–62. 33239350 10.1136/bjsports-2020-102955 PMC7719906

[32] Balady GJ, Arena R, Sietsema K, et al. Clinician’s guide to cardiopulmonary exercise testing in adults. Circulation. 2010;122(2):191–225. 20585013 10.1161/CIR.0b013e3181e52e69

[33] Imboden MT, Kaminsky LA, Peterman JE, et al. Cardiorespiratory fitness normalized to fat-free mass and mortality risk. Med Sci Sports Exerc. 2020;52(7):1532–7. 31985577 10.1249/MSS.0000000000002289

[34] Volaklis KA, Halle M, Meisinger C. Muscular strength as a strong predictor of mortality: a narrative review. Eur J Intern Med. 2015;26(5):303–10. 25921473 10.1016/j.ejim.2015.04.013

[35] Benjamini Y, Hochberg Y. Controlling the false discovery rate: a practical and powerful approach to multiple testing. J R Stat Soc Series B Stat Methodol. 1995;57(1):289–300.

[36] Bean JF, Kiely DK, LaRose S, Alian J, Frontera WR. Is stair climb power a clinically relevant measure of leg power impairments in at-risk older Aaults? Arch Phys Med Rehabil. 2007;88(5):604–9. 17466729 10.1016/j.apmr.2007.02.004

[37] Zanuso S, Sacchetti M, Sundberg CJ, Orlando G, Benvenuti P, Balducci S. Exercise in type 2 diabetes: genetic, metabolic and neuromuscular adaptations: a review of the evidence. Br J Sports Med. 2017;51(21):1533–8. 28501806 10.1136/bjsports-2016-096724

[38] Lafortuna CL, Agosti F, Marinone PG, Marazzi N, Sartorio A. The relationship between body composition and muscle power output in men and women with obesity. J Endocrinol Invest. 2004;27(9):854–61. 15648550 10.1007/BF03346280

[39] Houston DK, Ding J, Nicklas BJ, et al. Overweight and obesity over the adult life course and incident mobility limitation in older adults: the health, aging and body composition study. Am J Epidemiol. 2009;169(8):927–36. 19270048 10.1093/aje/kwp007 PMC2727232

[40] Sartorio A, Lafortuna CL, Agosti F, Proietti M, Maffiuletti NA. Elderly obese women display the greatest improvement in stair climbing performance after a 3-week body mass reduction program. Int J Obes Relat Metab Disord. 2004;28(9):1097–104. 15211371 10.1038/sj.ijo.0802702

[41] Ettinger WH Jr, Burns R, Messier SP, et al. A randomized trial comparing aerobic exercise and resistance exercise with a health education program in older adults with knee osteoarthritis: the Fitness Arthritis and Seniors Trial (FAST). JAMA. 1997;277(1):25–31. 8980206

[42] Nicklas BJ, Chmelo E, Delbono O, Carr JJ, Lyles MF, Marsh AP. Effects of resistance training with and without caloric restriction on physical function and mobility in overweight and obese older adults: a randomized controlled trial. Am J Clin Nutr. 2015;101(5):991–9. 25762810 10.3945/ajcn.114.105270 PMC4409692

[43] Wilding JPH, Batterham RL, Calanna S, et al. Once-weekly semaglutide in adults with overweight or obesity. N Engl J Med. 2021;384(11):989–1002. 33567185 10.1056/NEJMoa2032183

[44] Jastreboff AM, Aronne LJ, Ahmad NN, et al. Tirzepatide once weekly for the treatment of obesity. N Engl J Med. 2022;387(3):205–16. 35658024 10.1056/NEJMoa2206038

[45] Packer M, Zile MR, Kramer CM, et al. Tirzepatide for heart failure with preserved ejection fraction and obesity. N Engl J Med. 2025;392(5):427–37. 39555826 10.1056/NEJMoa2410027

[46] Kosiborod MN, Abildstrøm SZ, Borlaug BA, et al. Semaglutide in patients with heart failure with preserved ejection fraction and obesity. N Engl J Med. 2023;389(12):1069–84. 37622681 10.1056/NEJMoa2306963

[47] Jorsal A, Kistorp C, Holmager P, et al. Effect of liraglutide, a glucagon-like peptide-1 analogue, on left ventricular function in stable chronic heart failure patients with and without diabetes (LIVE)-a multicentre, double-blind, randomised, placebo-controlled trial. Eur J Heart Fail. 2017;19(1):69–77. 27790809 10.1002/ejhf.657

[48] Osman AF, Mehra MR, Lavie CJ, Nunez E, Milani RV. The incremental prognostic importance of body fat adjusted peak oxygen consumption in chronic heart failure. J Am Coll Cardiol. 2000;36(7):2126–31. 11127451 10.1016/s0735-1097(00)00985-2

[49] Krachler B, Savonen K, Komulainen P, Hassinen M, Lakka TA, Rauramaa R. Cardiopulmonary fitness is a function of lean mass, not total body weight: the DR’s EXTRA study. Eur J Prev Cardiol. 2020;22(9):1171–9. 10.1177/2047487314557962 25381337

[50] Lolli L, Batterham AM, Weston KL, Atkinson G. Size exponents for scaling maximal oxygen uptake in over 6500 humans: a systematic review and meta-analysis. Sports Med. 2017;47(7):1405–19. 28058696 10.1007/s40279-016-0655-1

[51] Bassett JDR, Howley ET. Limiting factors for maximum oxygen uptake and determinants of endurance performance. Med Sci Sports Exerc. 2000;32(1):70–84. 10647532 10.1097/00005768-200001000-00012

[52] Donini LM, Busetto L, Bischoff SC, et al. Definition and diagnostic criteria for sarcopenic obesity: ESPEN and EASO consensus statement. Obes Facts. 2022;5(3):321–35. 10.1159/000521241 PMC9210010 35196654

[53] Villareal DT, Aguirre L, Gurney AB, et al. Aerobic or resistance exercise, or both, in dieting obese older adults. N Engl J Med. 2017;376(20):1943–55. 28514618 10.1056/NEJMoa1616338 PMC5552187

[54] Donnelly JE, Blair SN, Jakicic JM, Manore MM, Rankin JW, Smith BK. American College of Sports Medicine position stand. Appropriate physical activity intervention strategies for weight loss and prevention of weight regain for adults. Med Sci Sports Exerc. 2009;41(2):459–71. 19127177 10.1249/MSS.0b013e3181949333

[55] Johnson NA, Sultana RN, Brown WJ, Bauman AE, Gill T. Physical activity in the management of obesity in adults: a position statement from Exercise and Sport Science Australia. J Sci Med Sport. 2021;24(12):1245–54. 34531124 10.1016/j.jsams.2021.07.009

[56] Jakicic JM, Powell KE, Campbell WW, et al. Physical activity and the prevention of weight gain in adults: a systematic review. Med Sci Sports Exerc. 2019;51(6):1262–9. 31095083 10.1249/MSS.0000000000001938 PMC6527311

