# Supplementary material for: Physical Fitness with Exercise and GLP-1 Receptor Agonist Treatment Alone or Combined After Diet-Induced Weight Loss: A Secondary Analysis of a Randomized Controlled Trial in Adults with Obesity
Source: Sports Med. 2026 Jan 24;56(7):1785–800. doi: 10.1007/s40279-025-02386-0 (PMC13388483; doi:10.1007/s40279-025-02386-0)
Supplement: Supplementary file 1 — Supplementary file1 (PDF 812 KB) [file 40279_2025_2386_MOESM1_ESM.pdf]

SUPPLEMENTARY FILE

Physical fitness with exercise and GLP-1 receptor agonist treatment alone or combined after diet-induced weight loss: a secondary analysis of a randomized controlled trial in adults with obesity

Authors list

Simon Birk Kjær Jensen, Matteo Fiorenza, Christian Rimer Juhl, Rasmus Michael Sandsdal, Emma Jensen, Søren Sonnenborg Seier, Charlotte Janus, Julie Rehné Jørgensen, Martin Bæk Blond, Jens Juul Holst, Bente Merete Stallknecht, Sten Madsbad, Thomas Bandholm, Signe Sørensen Torekov

Contents

Figure S1. Flow chart 3
Figure S2. Box plots and individual participant data for changes in physical fitness outcomes ... 4
Table S1. Physical fitness construct framework 5
Table S2. Key secondary and supportive secondary outcomes – per-protocol analysis 6
Table S3. Estimated between-group differences for supportive secondary outcomes 7

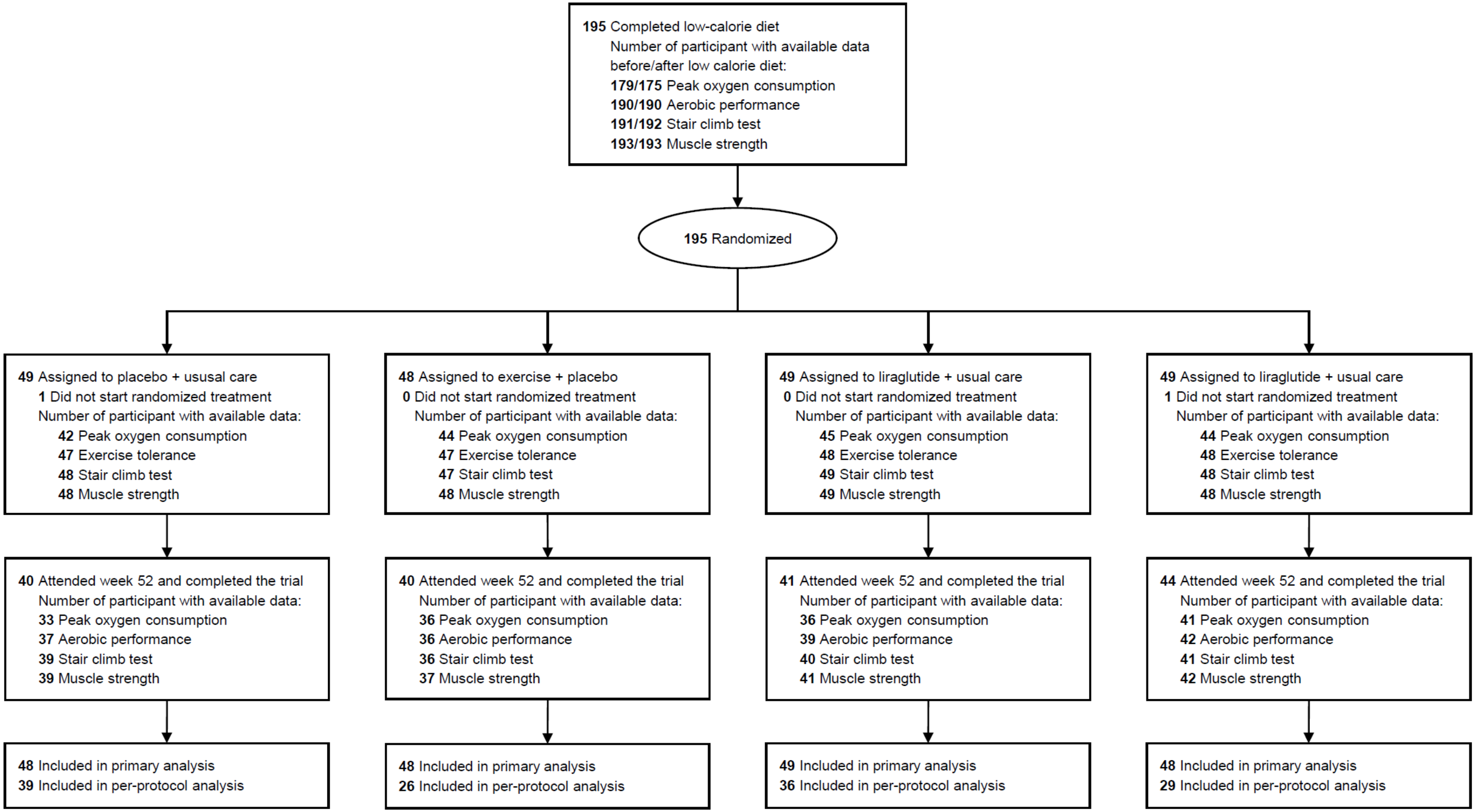

Figure S1. Flow chart

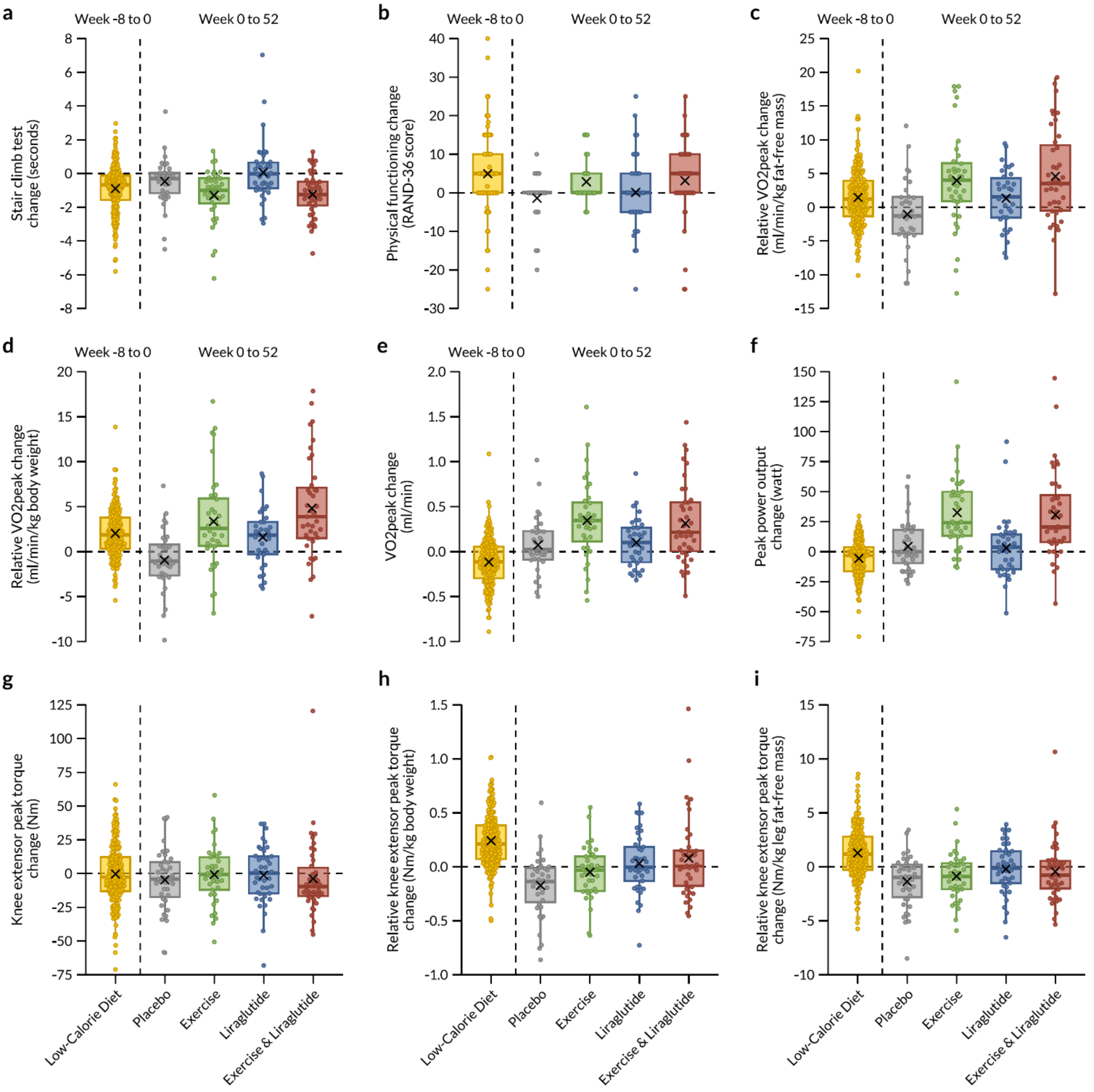

Figure S2. Box plots and individual participant data for changes in physical fitness outcomes
Observed changes in outcomes related to physical functioning (**a-b**), cardiorespiratory fitness (**c-f**), and muscle strength (**g-i**). Data are presented as changes in response to the low-calorie diet for all participants (week -8 to 0) and from randomization (week 0) to week 52 for the four randomized treatment groups. In the box plots, the lines inside the boxes indicate medians; the box the interquartile range (from Q1 to Q3); the cross means; and the whiskers smallest and largest values within 1.5 x the interquartile range. Dots indicate individual participant values. **a**, Change in stair climb test performance. **b**, Change in physical functioning score from the RAND-36 questionnaire. **c**, Change in $\dot{V}O_{2peak}$ relative to fat-free mass. **d**, Change in $\dot{V}O_{2peak}$ relative to body weight. **e**, Change in absolute $\dot{V}O_{2peak}$. **f**, Change in peak power output. **g**, Change in knee extensor peak torque. **h**, Change in knee extensor peak torque relative to body weight. **i**, Change in knee extensor peak torque relative to leg fat-free mass.

Table S1. Physical fitness construct framework	
**Term**	**Definition**
Physical fitness	The ability to carry out daily tasks and perform physical activities in a highly functional state, often as a result of physical conditioning.
**Specific physical fitness constructs**	
Physical functioning	The ability to perform basic and instrumental activities of daily living, divided into four subdomains: mobility (lower limbs); dexterity (upper limbs); axial or central functions (neck and back); and complex activities related to instrumental activities of daily living.
Physical functional performance	The ability to perform measurable physical tasks that reflect real-world functional activities, ranging from basic to complex movements (e.g., walking, climbing stairs, or carrying objects).
Cardiorespiratory fitness	A measure of the functional capabilities of the heart, lungs and muscles, relative to the demands of specific exercise routines such as running or cycling.
Muscle strength	The amount of force generated by muscle contraction.

Table S2. Key secondary and supportive secondary outcomes – per-protocol analysis

	Placebo	Exercise	Liraglutide	Exercise and liraglutide
**KEY SECONDARY OUTCOMES**				
**Physical functional performance (stair climb test performance), s**				
Change from week 0 to 52	-0.4 (-0.9; 0.0)	-1.5 (-2.0; -0.9)	0.0 (-0.5; 0.5)	-1.6 (-2.1; -1.0)
ETD vs. placebo	*NA*	-1.0 (-1.8; -0.3)[a]	0.4 (-0.3; 1.1)	-1.1 (-1.9; -0.4)[a]
ETD vs. exercise	*NA*	*NA*	1.5 (0.7; 2.2)[b]	-0.1 (-0.9; 0.7)
ETD vs. liraglutide	*NA*	*NA*	*NA*	-1.6 (-2.3; -0.8)[b]
**Cardiorespiratory fitness ($\dot{V}O2_{peak}$ normalized to FFM), ml/min/kg FFM**				
Change from week 0 to 52	-1.2 (-3.1; 0.7)	6.7 (4.5; 8.8)	1.0 (-0.9; 3.0)	5.8 (3.7; 7.8)
ETD vs. placebo	*NA*	7.9 (5.0; 10.7)[a]	2.2 (-0.4; 4.9)	7.0 (4.2; 9.7)[a]
ETD vs. exercise	*NA*	*NA*	-5.6 (-8.5; -2.8)[b]	-0.9 (-3.8; 2.0)
ETD vs. liraglutide	*NA*	*NA*	*NA*	4.7 (2.0; 7.5)[b]
**Muscle strength (isometric knee extensor peak torque), Nm**				
Change from week 0 to 52	-4.7 (-12.2; 2.7)	2.5 (-6.5; 11.6)	-3.0 (-10.7; 4.8)	-6.6 (-15.3; 2.2)
ETD vs. placebo	*NA*	7.3 (-4.3; 18.8)	1.8 (-8.8; 12.3)	-1.9 (-13.2; 9.5)
ETD vs. exercise	*NA*	*NA*	-5.5 (-17.3; 6.3)	-9.1 (-21.6; 3.4)
ETD vs. liraglutide	*NA*	*NA*	*NA*	-3.6 (-15.1; 7.9)
**SUPPORTIVE SECONDARY OUTCOMES (within-group changes from randomization (week 0) to week 52)**				
**$\dot{V}O2_{peak}$, ml/min/ kg BW**	-1.0 (-2.5; 0.5)	5.3 (3.6; 7)[a,b]	1.2 (-0.3; 2.7)[a]	5.7 (4.1; 7.3)[a,b]
**$\dot{V}O2_{peak}$, ml/min**	70 (-52; 191)	479 (342; 617)[a,b]	57 (-67; 182)	374 (244; 505)[a,b]
**Peak power output, W**	6.2 (-2.3; 14.7)	44.2 (34.0; 54.5)[a,b]	3.0 (-6.0; 12.0)	38.1 (28.4; 47.8)[a,b]
**Peak Torque, Nm/kg leg FFM**	-1.4 (-2.2; -0.7)	-0.5 (-1.3; 0.4)	-0.3 (-1.1; 0.5)[a]	-0.6 (-1.4; 0.3)
**Peak Torque, Nm/kg BW**	-0.2 (-0.3; -0.1)	0.0 (-0.1; 0.1)[a]	0.0 (-0.1; 0.1)[a]	0.1 (0.0; 0.2)[a]
**Physical functioning score[c]**	-0.9 (-3.3; 1.5)	3.0 (0.0; 5.9)[a]	0.2 (-2.3; 2.7)	4.2 (1.5; 7.0)[a,b]
**Appendicular FFM, kg**	1.36 (0.88; 1.84)	1.23 (0.66; 1.81)[b]	0.08 (-0.42; 0.58)[a]	-0.11 (-0.66; 0.44)[a]
**Appendicular FFM, kg/kg BW*100**	-0.35 (-0.87; 0.17)	1.1 (0.46; 1.73)[a]	0.64 (0.11; 1.18)[a]	1.76 (1.16; 2.36)[a,b]

Values are mean (95% CI) changes from randomization (week 0) to week 52 estimated from a constrained linear mixed model in the per-protocol population (n=130). The model was specified with inherent pre-randomization adjustment and the following fixed effects: time, time-treatment interaction, sex, and age group.
For the three key secondary outcomes, estimated treatment differences (ETD) with 95% CI are reported as between-group differences.
[a] The 95% CI of the comparison to placebo does not include 0, indicating a statistically significant difference.
[b] The 95% CI of the comparison to liraglutide does not include 0, indicating a statistically significant difference.
[c] Physical functioning score from the RAND 36-item health survey ranging from 0-100 with higher scores indicating better physical functioning.
Abbreviations: $\dot{V}O2_{peak}$, peak oxygen consumption; FFM, fat-free mass; BW, body weight; Nm, newton meter; ETD, estimated treatment difference.

Table S3. Estimated between-group differences for supportive secondary outcomes						
	Exercise vs. placebo	Liraglutide vs. placebo	Exercise and liraglutide vs. placebo	Liraglutide vs. exercise	Exercise and liraglutide vs. exercise	Exercise and liraglutide vs. liraglutide
**$\dot{V}O2_{peak}$, ml/min**	6.1 (3.5; 8.7)	2.3 (-0.4; 4.9)	5.3 (2.7; 7.8)	-3.8 (-6.4; -1.2)	-0.8 (-3.3; 1.7)	3.0 (0.5; 5.5)
**$\dot{V}O2_{peak}$, ml/min/kg BW**	4.8 (2.7; 6.9)	2.2 (0.1; 4.3)	5.3 (3.3; 7.4)	-2.6 (-4.7; -0.6)	0.5 (-1.5; 2.5)	3.2 (1.2; 5.2)
**Peak power output, W**	27.2 (14.9; 39.5)	-3.9 (-16.1; 8.2)	21.9 (10.0; 33.8)	-31.1 (-43.4; -18.9)	-5.3 (-17.2; 6.7)	25.9 (14.1; 37.6)
**Peak Torque, Nm/kg leg FFM**	0.84 (-0.15; 1.82)	1.07 (0.11; 2.04)	0.87 (-0.09; 1.82)	0.24 (-0.74; 1.22)	0.03 (-0.94; 1.00)	-0.21 (-1.15; 0.74)
**Peak Torque, Nm/kg BW**	0.16 (0.03; 0.29)	0.19 (0.06; 0.31)	0.24 (0.11; 0.36)	0.03 (-0.10; 0.16)	0.08 (-0.05; 0.20)	0.05 (-0.07; 0.17)
**Physical functioning score[d]**	2.6 (-0.9; 6.1)	0.6 (-2.8; 4.0)	4.1 (0.7; 7.5)	-2.0 (-5.5; 1.4)	1.5 (-1.9; 4.9)	3.5 (0.2; 6.8)
**Appendicular FFM, kg**	-0.32 (-0.98; 0.34)	-1.25 (-1.89; -0.61)	-1.24 (-1.88; -0.61)	-0.93 (-1.58; -0.28)	-0.93 (-1.57; -0.28)	0.00 (-0.62; 0.63)
**Appendicular FFM, kg/kg BW*100**	1.11 (0.32; 1.9)	0.91 (0.14; 1.68)	1.75 (0.99; 2.52)	-0.20 (-0.98; 0.59)	0.65 (-0.13; 1.42)	0.85 (0.09; 1.6)

Values are estimated mean (95% CI) between-group differences at week 52 in the full analysis set estimated from constrained linear mixed models with inherent pre-randomization adjustment and the following fixed effects: time, time-treatment interaction, sex, and age group.
Abbreviations: $\dot{V}O2_{peak}$, peak oxygen consumption; FFM, fat-free mass; BW, body weight; Nm, newton meter.
