# Supplementary material for: Physical Fitness with Exercise and GLP-1 Receptor Agonist Treatment Alone or Combined After Diet-Induced Weight Loss: A Secondary Analysis of a Randomized Controlled Trial in Adults with Obesity
Source: Sports Med. 2026 Jan 24;56(7):1785–800. doi: 10.1007/s40279-025-02386-0 (PMC13388483; doi:10.1007/s40279-025-02386-0)
Supplement: Supplementary file 2 — Supplementary file2 (PDF 822 KB) [file 40279_2025_2386_MOESM2_ESM.pdf]

Title page

Statistical Analysis Plan

Journal

Sports Medicine

Article title

Physical fitness with exercise and GLP-1 receptor agonist treatment alone or combined after diet-induced weight loss: a secondary analysis of a randomized controlled trial in adults with obesity

Authors list

Simon Birk Kjær Jensen[1#], Matteo Fiorenza[1], Christian Rimer Juhl[1], Rasmus Michael Sandsdal[1], Emma Jensen[1], Søren Sonnenborg Seier[1], Charlotte Janus[1], Julie Rehné Jørgensen[1], Martin Bæk Blond[2], Jens Juul Holst[1,3], Bente Merete Stallknecht[1], Sten Madsbad[4,8], Thomas Bandholm[5,6,7,8], Signe Sørensen Torekov[1#]

Affiliations

[1] Department of Biomedical Sciences, Faculty of Health and Medical Sciences, University of Copenhagen, Copenhagen, Denmark

[2] Clinical and Translational Research, Steno Diabetes Center Copenhagen, Herlev, Denmark

[3] Novo Nordisk Foundation Center for Basic Metabolic Research, University of Copenhagen, Copenhagen, Denmark

[4] Department of Endocrinology, Copenhagen University Hospital – Amager and Hvidovre, Copenhagen, Denmark

[5] Physical Medicine & Rehabilitation Research-Copenhagen, Department of Physical and Occupational Therapy, Copenhagen University Hospital – Amager and Hvidovre, Hvidovre, Denmark

[6] Department of Clinical Research, Copenhagen University Hospital – Amager and Hvidovre, Hvidovre, Denmark

[7] Department of Orthopedic Surgery, Copenhagen University Hospital – Amager and Hvidovre, Hvidovre, Denmark

[8] Department of Clinical Medicine, University of Copenhagen, Copenhagen, Denmark

#Correspondence

Simon Birk Kjær Jensen, simon.jensen@sund.ku.dk; Signe Sørensen Torekov, torekov@sund.ku.dk.

This document is a supplement to the S-LiTE study protocol[1] and contains the statistical analysis plan (SAP) for the report on secondary outcomes related to physical fitness. The document follows the Guidelines for the Content of Statistical Analysis Plans in Clinical Trials.[2]

Section 1: Administrative Information

Title and trial registration

SAP Title

Weight maintenance with exercise, liraglutide, or combination treatment for physical fitness in people with obesity – Statistical analysis plan for physical fitness outcomes in the S-LiTE randomized controlled trial

Trial registration

EudraCT no: 2015-005585-32; ClinicalTrials.gov Identifier: NCT04122716

SAP version

Version: 1.0
Date: December 20, 2024

Protocol version

This document has been written based on information contained in the study protocol version 10, dated November 20, 2019

SAP revisions

Protocol version	Updated SAP version no.	Section number changed	Description of and reason for change	Date changed
(currently no revisions)				

Roles and responsibility

SAP authors

Simon Birk Kjær Jensen[1], Matteo Fiorenza[1], Martin Bæk Blond[2], Thomas Bandholm[3], Signe Sørensen Torekov[1]

Statistical advisor

Martin Bæk Blond

Statistical analysts

Simon Birk Kjær Jensen and Matteo Fiorenza

Sponsor-investigator

Signe Sørensen Torekov

Principal investigator

Sten Madsbad[4]

Affiliations

[1] Department of Biomedical Sciences, Faculty of Health and Medical Sciences, University of Copenhagen, Copenhagen, Denmark
[2] Clinical and Translational Research, Steno Diabetes Center Copenhagen, Herlev, Denmark
[3] Department of Clinical Research; Physical Medicine and Rehabilitation Research-Copenhagen (PMR-C), Department of Physical and Occupational Therapy; Department of Orthopedic Surgery - all Copenhagen University Hospital – Amager and Hvidovre, Denmark; Department of Clinical Medicine, University of Copenhagen, Denmark.
[4] Department of Endocrinology, Copenhagen University Hospital – Amager and Hvidovre, Copenhagen, Denmark

Signatures

I hereby declare that I have reviewed and approved the statistical analysis plan with the title: "Weight maintenance with exercise, liraglutide, or combination treatment for physical fitness in people with obesity – Statistical analysis plan for physical fitness outcomes in the S-LiTE randomized controlled trial"

Name	Role	Signature	Date
Simon Birk Kjær Jensen	SAP author and statistical analyst	Simon Jensen	03-01-25
Matteo Fiorenza	SAP author and statistical analyst	[illegible]	20-12-24
Martin Bæk Blond	SAP author and statistical advisor	[illegible]	20-12-24
Thomas Bandholm	SAP author	[illegible]	20/12-2024
Signe Sørensen Torekov	SAP author and sponsor-investigator	[illegible]	03-01-25
Sten Madsbad	Principal investigator	[illegible]	03-01-25

Contents

Section 1: Administrative Information ... 1
Title and trial registration ... 1
SAP version ... 1
Protocol version ... 1
SAP revisions ... 1
Roles and responsibility ... 2
Signatures ... 2
Section 2: Introduction ... 4
Background and rationale ... 4
Objectives ... 4
Hypothesis ... 4
Section 3: Study Methods ... 4
Trial design ... 4
Randomization ... 5
Sample size ... 5
Framework ... 6
Statistical interim analyses and stopping guidance ... 6
Timing of final analysis ... 6
Timing of outcome assessments ... 6
Section 4: Statistical Principles ... 6
Confidence intervals and P values ... 6
Adherence and protocol deviations ... 6
Analysis populations ... 7
Section 5: Trial Population ... 7
Screening data ... 7
Eligibility ... 7
Recruitment ... 7
Withdrawal/follow-up ... 7
Baseline characteristics ... 7
Section 6: Analysis ... 8
Outcome definitions ... 8
Analysis methods ... 10
Missing data ... 10
Additional analyses ... 10
Harms ... 10
Statistical software ... 10
References ... 10

Section 2: Introduction

Background and rationale

Low cardiorespiratory fitness, functional performance, and muscle strength are independent predictors of all-cause mortality.[3-5] Obesity is associated with low cardiorespiratory fitness and physical function,[6,7] but high cardiorespiratory fitness largely mitigate the cardiovascular risk associated with obesity.[8] Accordingly, current guidelines emphasize that improved physical fitness and function should be a focus in obesity management.[9]

While incretin-based medications, such as glucagon-like peptide-1 receptor agonists (GLP-1 RA), induce substantial weight loss, they also lead to significant reductions in fat-free mass,[10,11] raising concerns about potential negative effects on muscle function and physical performance.[12,13] Comprehensive assessments of fitness and muscle function are essential to determine the implications of fat-free mass loss and to guide obesity treatments that balance large weight loss with improved physical fitness and function while also preserving or improving muscle strength.

In the S-LiTE trial (*Synergy effect of the appetite hormone GLP-1 (LiragluTide) and Exercise on maintenance of weight loss and health after a low-calorie diet*), the combination of a GLP-1 RA and a supervised exercise program improved healthy weight maintenance more than either treatment alone after diet-induced weight loss.[14] However, the effects of these treatment strategies on cardiorespiratory fitness, functional performance, and muscle strength have not been investigated.

Objectives

The primary objective is to investigate the effects of exercise, GLP-1 RA, the combination of both, and placebo after a diet-induced weight loss on cardiorespiratory fitness, functional performance, and muscle strength. The secondary objective is to investigate if a dose-response relationship exists between exercise volume and improvements in physical fitness and maintenance of healthy body weight after weight loss.

Hypothesis

The hypothesis is that combining exercise with GLP-1 RA treatment improves physical fitness compared with GLP-1 RA alone and placebo. Second, that exercise alone improves physical fitness compared with GLP-1 RA alone and placebo.

Section 3: Study Methods

This is a study of secondary outcomes in the S-LiTE trial. The SAP will be uploaded before beginning statistical analyses. A detailed description of the trial design is available with the primary trial report[14] and published protocol paper.[1] In the following section, the overall trial design will be briefly summarized and the methods specifically related to the outcomes of this SAP will be described in detail.

Trial design

The trial is a randomized controlled trial with an initial weight loss phase before randomization. Participants were randomized after a low-calorie diet-induced weight loss (at least 5% weight loss) to either:

1) Placebo + usual physical activity (placebo group)
2) Placebo + exercise program (exercise group)
3) Liraglutide + usual physical activity (liraglutide group)
4) Liraglutide + exercise program (combination group)

Treatment allocation was a 1:1:1:1 ratio.

Randomization

Randomization was stratified according to sex (male/female) and age group (below 40 years / 40 years or older). Randomization was done according to a subject randomization list by a non-blinded study nurse who had no other roles in the study.

Sample size

Original sample size calculation

The sample size was calculated based on the primary outcome of the primary trial report, change in body weight from randomization (week 0) to week 52.[14] It was estimated that a sample size of 30 in each group would have 80% power to detect a difference in means between groups of 4 kg assuming a common standard deviation of 5.5 kg with a 0.05 two-sided significance level. Allowing for loss to follow-up and potentially larger than expected standard deviation, a total of 195 participants were randomized of which 166 attended end-of-treatment assessments at week 52 (40 of 49 (82%) in the placebo group, 40 of 48 (83%) in the exercise group, 41 of 49 (84%) in the liraglutide group, and 45 of 49 (92%) in the combination group.

We determined the minimum relevant differences in the three main outcomes related to physical fitness and calculated expected power (based on two-sample t-tests and 40 participants in each group) in relation to writing this statistical analysis plan (see below) after the last participant's last visit but before data was extracted for statistical analyses.

Cardiorespiratory fitness

A sample size of 40 in each group is expected to have 85% power to detect a difference in means of 3 ml/min/kg fat-free mass assuming that the common standard deviation for change is 4.4 ml/min/kg fat-free mass, with a 0.05 two-sided significance level. The estimate used for the standard deviation was based on previous interventions with weight loss, exercise, or both, in adults with overweight or obesity, and measured peak oxygen consumption normalized to fat-free mass.[15,16] An increase in cardiorespiratory fitness of 1 ml/min/kg fat-free mass is associated with reduced all-cause and cardiovascular mortality.[17] Exercise interventions in accordance with consensus recommendations typically show improvements in cardiorespiratory fitness of about 10% in sedentary adults, a magnitude which is associated with considerable improvements in survival.[3] We expect a 10% increase in cardiorespiratory fitness to correspond to 3-4 ml/min/kg based on previous studies with similar participant characteristics at baseline.[15,18,19] Thus, a between-group difference of 3 ml/min/kg fat-free mass is considered clinically relevant and realistic to detect and was therefore defined as the minimum relevant difference.

Functional performance (Stair climb test performance)

A sample size of 40 in each group is expected to have 89% power to detect a difference in means of 0.8 seconds in the stair climb test performance, assuming that the common standard deviation for change is 1.1 seconds.[20] The stair climb test that we used is not a standardized test. Therefore, estimates for mean difference and standard deviation are based on relative changes and the assumption that the stair climb test will take a mean 15-20 seconds to complete. This assumption was based on a previous study, in which the time to complete a stair climb test of half the distance, including only the ascending part, took 9 seconds on average.[20] Stair test performance is expected to decline about 0.8-1% per year in mid and late life,[7,21] indicating that a 5% improvement in stair climb performance corresponds to preventing the impact of 5 years of aging. Exercise interventions alone or as add-on to calorie restriction typically improves functional performance by 5-10%.[22-25] The participants in these studies were generally characterized by old age or presence of a comorbidity. We expect our study population to have higher baseline levels of functional performance, which could result in improvement in stair climb performance in the low range of this interval. Thus, a difference between groups in stair climb performance of 0.8 seconds, corresponding to ~5%, is considered clinically relevant and realistic to detect and was therefore defined as the minimum relevant difference.

Muscle strength (knee-extensor isometric strength)

A sample size of 40 in each group is expected to have 80% power to detect a difference in means of 15 newton meter (Nm) assuming that the common standard deviation for change is 23 Nm.[16] We expect a change of 15 Nm to correspond to a change of approximately 10 % in strength. A decrease in strength of this magnitude may be linked to decreased physical function and mortality[26-28] and was defined as the minimum relevant difference.

Framework

Superiority hypothesis testing framework. For the three main outcomes, the four groups will be compared with each other. Superiority will be claimed for a between-group difference if $P < 0.05$ for a hypothesis test controlled at a false discovery rate of 5%.

Statistical interim analyses and stopping guidance

No interim analysis were planned, and no stopping guidelines were made.

Timing of final analysis

All outcomes will be analyzed collectively after the last participant's last visit and after the statistical analysis plan has been signed by all SAP authors.

Timing of outcome assessments

All outcomes related to physical fitness were measured three times:

1) At study inclusion, i.e. before starting the low-calorie diet (week -8)
2) At randomization, i.e. after completing the low-calorie diet: (week 0)
3) At end-of-treatment, i.e. 52 weeks after randomization: (week 52)

Section 4: Statistical Principles

Confidence intervals and P values

All statistical analyses to test between-group differences for the three main fitness outcomes will be 2-sided and will be performed using a 5% significance level. The four intervention groups will be compared with each other. The Benjamini-Hochberg procedure[29] will be used to control the false discovery rate for tests related to the three main fitness outcomes. Accordingly, P values will be computed and arranged in ascending order. Each P value will be compared to its critical value, $\frac{i}{m}Q$, where i is the rank, m is the total number of tests, and Q is the false discovery rate (5%). The largest P value $\leq \frac{i}{m}Q$ will be considered statistically significant together with all smaller P values. The results of all other analyses of exploratory outcomes specified in this SAP will be reported with point estimates and confidence intervals unadjusted for multiple testing. All confidence intervals presented will be 95% and two-sided. 95% confidence intervals will be presented for within-group changes and between-group differences.

Adherence and protocol deviations

Adherence to treatment is based on medication dose and exercise volume from the time that treatment was fully up-titrated until week 52. Study adherence has been published in the primary trial report in tables S7 and S8.[14] Medication dose was noted 12 times in the intervention period. Exercise volume was recorded by sports watches and heart rate monitors. Time spent at different exercise intensities were calculated as: very light intensity (<57% of maximum heart rate), light intensity (57-63%), moderate intensity (64-76%), vigorous intensity (77-95%) and near-maximal to maximal intensity (≥ 96%).[30] Time spent doing moderate-to-vigorous intensity exercise will be calculated as minutes per week of all exercise with an intensity of at least 64% of maximum heart rate. Exercise adherence was calculated as percentage of WHO's global recommendations on physical activity for health (48): *Adults aged 18–64 should do at least 150 minutes of moderate-intensity aerobic physical activity throughout the week or do at least 75 minutes of vigorous-*

intensity aerobic physical activity throughout the week or an equivalent combination of moderate- and vigorous-intensity activity. As such, one minute of moderate intensity exercise accounted for 1/150 and one minute of vigorous or near-maximal intensity accounted for 1/75 of prescribed weekly exercise. Protocol deviators were defined as not fulfilling the criteria for per-protocol (see below).

Analysis populations

Full analysis set

All participants who underwent randomization and received at least one dose of randomized treatment (i.e., took at least one dose of study medication or completed at least one exercise session), irrespective of adherence to interventions and completion of the study (consistent with the modified intention to treat approach).

Per-protocol analysis set

All participants who completed the 52-week randomized treatment period with sufficient compliance to study medication and/or the exercise program, defined for the four interventions groups as:
Placebo group: Volume-matched placebo corresponding to 2.4-3.0 mg subcutaneous liraglutide once-daily for at least 75% of the intervention period after up titration.
Exercise group: Volume-matched placebo corresponding to 2.4-3.0 mg subcutaneous liraglutide once-daily for at least 75% of the intervention period after up titration and at least 75% of the WHO minimum recommendations on physical activity for health.
Liraglutide group: 2.4-3.0 mg subcutaneous liraglutide once daily for at least 75% of the intervention period after up titration.
Combination group: 2.4-3.0 mg subcutaneous liraglutide once daily for at least 75% of the intervention period after up titration and at least 75% of the WHO minimum recommendations on physical activity for health.

Section 5: Trial Population

Screening data

Screening data will not be reported.

Eligibility

Eligible participants were adults (age 18-65) with a BMI of 32 to 43 kg/m^2. A full list of inclusion and exclusion criteria have been published.[1,14]

Recruitment

A CONSORT flow diagram will be completed and will summarize the number of participants who: Initiated low-calorie diet; completed low-calorie diet; were randomized to treatment; received at least one dose of randomized intervention; attended body weight measurement at week 52 (the primary outcomes of the primary trial); completed fitness tests before and after low-calorie diet and at week 52; did not complete/had invalid fitness tests.

Withdrawal/follow-up

All randomized participants who discontinued treatment were kept in the study and invited for outcomes assessments at week 52, unless they withdrew consent. The numbers (with reasons) of missingness over the course of the intervention period was summarized for each treatment group in the CONSORT flow diagram in the primary trial report, figure S1.[14]

Baseline characteristics

Study participants will be described at inclusion/randomization regarding the following:

- Number of participants (men/women)

- Age (years)
- Body weight (kg)
- BMI (kg/m^2)
- Whole-body fat mass (kg)
- Whole-body fat free mass (kg)
- Whole-body fat percentage (%)
- Waist circumference (cm)
- Peak oxygen consumption in absolute values (ml/min) and normalized to fat-free mass and body weight (ml/min/kg)
- Time to exhaustion during the incremental cycling exercise test (min:sec)
- Peak power output during the incremental cycling exercise test (W) and normalized to body weight (W/kg)
- Oxygen consumption at submaximal loads (ml/min and ml/min/kg)
- Stair climb test (seconds to complete the test)
- Maximum isometric knee extensor strength in absolute values (Nm) and normalized to body weight and leg fat-free mass (Nm/kg)
- Appendicular fat-free mass in absolute values (kg) and normalized to body weight (kg/kg*100)

The distribution of all continuous data included in baseline characteristics will be visually inspected using QQ-plots and histograms. Continuous data will be summarized by mean and standard deviation if data is normal distributed and median and interquartile range if data is not normal distributed. Categorical data will be summarized by numbers and percentages.

Section 6: Analysis

Outcome definitions

Main outcomes

<u>Peak oxygen uptake normalized to fat-free mass (ml/min/kg fat-free mass) from randomization to end of treatment (week 0 to 52).</u>

Cardiorespiratory fitness will be measured as the peak oxygen uptake ($\dot{V}O_2$peak) during an incremental test to exhaustion on an electromagnetically braked cycle ergometer (Corival, Lode Medical Technology, The Netherlands). Pulmonary oxygen uptake ($\dot{V}O_2$) and carbon dioxide production ($\dot{V}CO_2$) were measured breath-by-breath using an online gas analysis system (MasterScreen CPX, CareFusion, Germany). A differentiated incremental test protocol was applied for females and males. The incremental test began with a 4-min submaximal bout (40 W for females and 50 W for males) followed by another 4-min submaximal bout at a higher workload (80 W for females and 100 W for males). Subsequently, the workload was stepwise increased (20 watts for females and 25 W for males) every minute until volitional exhaustion. Cadence was kept at 70-90 revolutions per minute throughout the text. Peak oxygen uptake was calculated as the average of the three highest $\dot{V}O_2$ measurements. Assessment of peak oxygen consumption was considered valid if the participant completed at least the first 8 minutes and reached a respiratory exchange ratio ($\dot{V}CO_2/\dot{V}O_2$) ≥ 1.1.[31] Participants were verbally encouraged to cycle for as long as possible. To account for differences in body size, peak oxygen consumption will be normalized to fat-free mass for the following reasons: 1) it is less biased in people with obesity in contrast to total body weight, 2) oxygen consumption is directly related to fat-free mass, 3) it is suggested to better reflect cardiorespiratory fitness as related to exercise performance and aerobic function, 4) it is associated with reduced mortality with higher prognostic value than normalization to body weight.[17,32-35]

<u>Stair climb test performance (seconds) from randomization to end of treatment (week 0 to 52).</u>

Overall functional performance will be measured as the time (seconds) to ascend and descend an 11-step (17 cm rise, 27 cm run) stairway twice. There was a ~2×2 m platform at the top and

bottom. The test began with participants standing with both feet pointing towards the stairs. When they were told to begin, they climbed to the top platform (both feet on the platform), climbed down to the lower platform, and repeated once. Participants were instructed to complete the test as fast as possible. The use of handrails for balance support was allowed if needed. The participants were given the option of one practice try before the test. Stair climb tests have been used frequently to assess physical function, also in populations with obesity.[22,23,36] In addition, the test reflects anaerobic power, lower limb strength, balance, and mobility. We considered this test to be relevant in the study population because stair climb tests are sensitive to detect declines in physical functioning in mid/late life and high BMI has been shown to accelerate this decline and increase the risk of mobility limitations.[6,7,21]

<u>Knee extensor strength (Nm) from randomization to end of treatment (week 0 to 52).</u>
Muscle strength will be determined as knee extensor peak torque (Nm) during an isometric maximal voluntary contraction. Force will be measured using a Good Strength Metitur dynamometer chair (Metitur Oy, Jyväskylä, Finland). Knee extensor peak force will be determined as the highest value obtained during 5 maximal isometric contractions of ~5 sec duration separated by 60 seconds rest. The test was performed on the dominant leg (participants were asked: *if you were to kick a ball, what foot would you use?*). The leg was fixed in a 60° knee flexion angle using a goniometer with the fixed arm pointed toward the greater trochanter, goniometer center mark at the lateral knee joint line, and movable goniometer arm pointed towards the lateral malleolus. The leg was placed in the transducer support with transducer center 8 cm above lateral malleolus. During the tests, participants sat with arms crossed and hands placed on contralateral shoulders. Participants were instructed to slowly increase force output and reach maximal effort after ~3 sec. Standard maximal effort encouragement was given during testing. The lever arm was measured as the length from the lateral epicondyle to the center of the leg-supporting chalice. Peak torque was measured as peak force multiplied by the lever arm.

Exploratory outcomes
<u>Outcomes related to cardiorespiratory fitness and aerobic performance:</u>

- Peak oxygen consumption in absolute values (ml/min) and normalized to body weight (ml/min/kg)
- Time to exhaustion during the incremental cycling exercise test (min:sec)
- Peak power output during the incremental cycling exercise test in absolute values (W) and normalized to body weight (W/kg)
- Oxygen consumption at submaximal loads (ml/min and ml/min/kg)

<u>Outcomes related to functional performance:</u>

- Physical function (RAND 36-Item Short Form Health Survey)
- Role limitations due to physical health (RAND 36-Item Short Form Health Survey)

<u>Outcomes related to muscle strength:</u>

- Knee extensor peak torque normalized to body weight (Nm/kg) and to leg fat-free mass (Nm/kg) as a measure of muscle quality

<u>Outcomes related to body composition:</u>

- Appendicular fat-free mass in absolute values (kg) and normalized to body weight (kg/kg*100)

<u>Outcomes related to dose-response relationship between exercise dose and fitness/body composition outcomes:</u>
Relationship between moderate-to-vigorous intensity exercise minutes (average from week 7 to 52) and changes from randomization to end of treatment (week 0 to 52) in the following:

- Peak oxygen consumption (ml/min/kg fat-free mass)
- Stair climb test (seconds)
- Knee extensor peak torque (Nm)
- Body weight (kg)

- Whole-body-fat percentage (percentage-points)
- Whole-body fat mass (kg)
- Whole-body fat-free mass (kg)
- Appendicular fat-free mass (kg)

Analysis methods

The primary analysis of the three main fitness outcomes will be performed on the full analysis set. Outcomes will be analyzed using constrained linear mixed models with inherent pre-randomization adjustment by putting all participants in the placebo group at week -8 and 0 (low-calorie diet).[37,38] The model will include time (factorial), a time-treatment interaction, sex (male, female), and age group (<40 years, ≥40 years) as fixed effects and will be specified with an unstructured covariance pattern to account for repeated measurements on each participant. Adequacy of model assumptions will be assessed using graphical models, and outcome variables may be logarithmically transformed if considered necessary to meet the assumptions of variance homogeneity and normality of residuals. Estimated mean differences between groups in the three main outcomes will be null hypothesis tested and reported with P values and 95% CI. A per protocol supplementary analysis will be performed for the main outcomes, excluding all protocol deviators. All exploratory outcomes will be analyzed using a similar statistical model and reported as estimated within-group changes and between-group differences with 95% CI.

Multiple linear regression analyses will be done to assess whether a dose-response relationship exists between exercise volume (average minutes per week of moderate-to-vigorous intensity exercise) and changes in body composition and physical fitness outcomes. Sex, age, liraglutide (yes/no) and outcome value at week 0 will be included as fixed effects. The analyses will include all participants randomized to exercise (exercise group and combination group).

Missing data

The number/frequency of missing values for the main outcomes will be provided for each group at each time point in the CONSORT flow diagram. Missing data will be assumed to be missing at random and handled implicitly by maximum likelihood estimation in the constrained linear mixed model.

Additional analyses

Not relevant.

Harms

All harms in the study has been published and is available in tables 2, S11, and S12 of the primary trial report.[14]

Statistical software

R version 3.6.0 or newer (The R Foundation for Statistical Computing, www.R-project.org) and SAS version 9.4 or newer (SAS Institute, Cary, NC, USA).

References

1. Jensen SBK, Lundgren JR, Janus C, Juhl CR, Olsen LM, Rosenkilde M, Holst JJ, Stallknecht BM, Madsbad S, Torekov SS. Protocol for a randomised controlled trial of the combined effects of the GLP-1 receptor agonist liraglutide and exercise on maintenance of weight loss and health after a very low-calorie diet. *BMJ Open*. 2019;9:e031431. doi: 10.1136/bmjopen-2019-031431
2. Gamble C, Krishan A, Stocken D, Lewis S, Juszczak E, Doré C, Williamson PR, Altman DG, Montgomery A, Lim P, et al. Guidelines for the Content of Statistical Analysis Plans in Clinical Trials. *Jama*. 2017;318:2337-2343. doi: 10.1001/jama.2017.18556

3. Ross R, Blair SN, Arena R, Church TS, Després J-P, Franklin BA, Haskell WL, Kaminsky LA, Levine BD, Lavie CJ, et al. Importance of Assessing Cardiorespiratory Fitness in Clinical Practice: A Case for Fitness as a Clinical Vital Sign: A Scientific Statement From the American Heart Association. *Circulation*. 2016;134:e653-e699. doi: 10.1161/cir.0000000000000461
4. García-Hermoso A, Cavero-Redondo I, Ramírez-Vélez R, Ruiz JR, Ortega FB, Lee DC, Martínez-Vizcaíno V. Muscular Strength as a Predictor of All-Cause Mortality in an Apparently Healthy Population: A Systematic Review and Meta-Analysis of Data From Approximately 2 Million Men and Women. *Arch Phys Med Rehabil*. 2018;99:2100-2113.e2105. doi: 10.1016/j.apmr.2018.01.008
5. Molari M, Fernandes KBP, Marquez AdS, Probst VS, Bignardi PR, Teixeira DdC. Impact of physical and functional fitness on mortality from all causes of physically independent older adults. *Archives of Gerontology and Geriatrics*. 2021;97:104524. doi: https://doi.org/10.1016/j.archger.2021.104524
6. Houston DK, Ding J, Nicklas BJ, Harris TB, Lee JS, Nevitt MC, Rubin SM, Tylavsky FA, Kritchevsky SB, Study ftHA. Overweight and Obesity Over the Adult Life Course and Incident Mobility Limitation in Older Adults: The Health, Aging and Body Composition Study. *American Journal of Epidemiology*. 2009;169:927-936. doi: 10.1093/aje/kwp007
7. Lange-Maia BS, Karvonen-Gutierrez CA, Strotmeyer ES, Avery EF, Appelhans BM, Fitzpatrick SL, Janssen I, Dugan SA, Kravitz HM. Factors Influencing Longitudinal Stair Climb Performance from Midlife to Early Late Life: The Study of Women's Health Across the Nation Chicago and Michigan Sites. *J Nutr Health Aging*. 2019;23:821-828. doi: 10.1007/s12603-019-1254-2
8. Oktay AA, Lavie CJ, Kokkinos PF, Parto P, Pandey A, Ventura HO. The Interaction of Cardiorespiratory Fitness With Obesity and the Obesity Paradox in Cardiovascular Disease. *Progress in Cardiovascular Diseases*. 2017;60:30-44. doi: https://doi.org/10.1016/j.pcad.2017.05.005
9. Busetto L, Dicker D, Frühbeck G, Halford JCG, Sbraccia P, Yumuk V, Goossens GH. A new framework for the diagnosis, staging and management of obesity in adults. *Nature Medicine*. 2024;30:2395-2399. doi: 10.1038/s41591-024-03095-3
10. Wilding JPH, Batterham RL, Calanna S, Davies M, Van Gaal LF, Lingvay I, McGowan BM, Rosenstock J, Tran MTD, Wadden TA, et al. Once-Weekly Semaglutide in Adults with Overweight or Obesity. *N Engl J Med*. 2021;384:989-1002. doi: 10.1056/NEJMoa2032183
11. McCrimmon RJ, Catarig A-M, Frias JP, Lausvig NL, le Roux CW, Thielke D, Lingvay I. Effects of once-weekly semaglutide vs once-daily canagliflozin on body composition in type 2 diabetes: a substudy of the SUSTAIN 8 randomised controlled clinical trial. *Diabetologia*. 2020;63:473-485. doi: 10.1007/s00125-019-05065-8
12. Mechanick JI, Butsch WS, Christensen SM, Hamdy O, Li Z, Prado CM, Heymsfield SB. Strategies for minimizing muscle loss during use of incretin-mimetic drugs for treatment of obesity. *Obes Rev*. 2024:e13841. doi: 10.1111/obr.13841
13. Hope DCD, Tan TM. Skeletal muscle loss and sarcopenia in obesity pharmacotherapy. *Nat Rev Endocrinol*. 2024;20:695-696. doi: 10.1038/s41574-024-01041-4
14. Lundgren JR, Janus C, Jensen SBK, Juhl CR, Olsen LM, Christensen RM, Svane MS, Bandholm T, Bojsen-Møller KN, Blond MB, et al. Healthy Weight Loss Maintenance with Exercise, Liraglutide, or Both Combined. *N Engl J Med*. 2021;384:1719-1730. doi: 10.1056/NEJMoa2028198
15. Straznicky NE, Lambert EA, Nestel PJ, McGrane MT, Dawood T, Schlaich MP, Masuo K, Eikelis N, de Courten B, Mariani JA, et al. Sympathetic neural adaptation to hypocaloric diet with or without exercise training in obese metabolic syndrome subjects. *Diabetes*. 2010;59:71-79. doi: 10.2337/db09-0934

16. Weiss EP, Jordan RC, Frese EM, Albert SG, Villareal DT. Effects of Weight Loss on Lean Mass, Strength, Bone, and Aerobic Capacity. *Med Sci Sports Exerc*. 2017;49:206-217. doi: 10.1249/mss.0000000000001074
17. Imboden MT, Kaminsky LA, Peterman JE, Hutzler HL, Whaley MH, Fleenor BS, Harber MP. Cardiorespiratory Fitness Normalized to Fat-Free Mass and Mortality Risk. *Med Sci Sports Exerc*. 2020;52:1532-1537. doi: 10.1249/mss.0000000000002289
18. Arad AD, DiMenna FJ, Thomas N, Tamis-Holland J, Weil R, Geliebter A, Albu JB. High-intensity interval training without weight loss improves exercise but not basal or insulin-induced metabolism in overweight/obese African American women. *J Appl Physiol (1985)*. 2015;119:352-362. doi: 10.1152/japplphysiol.00306.2015
19. Trilk JL, Singhal A, Bigelman KA, Cureton KJ. Effect of sprint interval training on circulatory function during exercise in sedentary, overweight/obese women. *Eur J Appl Physiol*. 2011;111:1591-1597. doi: 10.1007/s00421-010-1777-z
20. Vincent KR, Braith RW, Feldman RA, Magyari PM, Cutler RB, Persin SA, Lennon SL, Gabr AH, Lowenthal DT. Resistance exercise and physical performance in adults aged 60 to 83. *J Am Geriatr Soc*. 2002;50:1100-1107. doi: 10.1046/j.1532-5415.2002.50267.x
21. Lafortuna CL, Agosti F, Marinone PG, Marazzi N, Sartorio A. The relationship between body composition and muscle power output in men and women with obesity. *J Endocrinol Invest*. 2004;27:854-861. doi: 10.1007/bf03346280
22. Sartorio A, Lafortuna CL, Agosti F, Proietti M, Maffiuletti NA. Elderly obese women display the greatest improvement in stair climbing performance after a 3-week body mass reduction program. *Int J Obes Relat Metab Disord*. 2004;28:1097-1104. doi: 10.1038/sj.ijo.0802702
23. Villareal DT, Chode S, Parimi N, Sinacore DR, Hilton T, Armamento-Villareal R, Napoli N, Qualls C, Shah K. Weight Loss, Exercise, or Both and Physical Function in Obese Older Adults. *New England Journal of Medicine*. 2011;364:1218-1229. doi: 10.1056/nejmoa1008234
24. Ettinger WH, Jr, Burns R, Messier SP, Applegate W, Rejeski WJ, Morgan T, Shumaker S, Berry MJ, O'Toole M, Monu J, et al. A Randomized Trial Comparing Aerobic Exercise and Resistance Exercise With a Health Education Program in Older Adults With Knee Osteoarthritis: The Fitness Arthritis and Seniors Trial (FAST). *JAMA*. 1997;277:25-31. doi: 10.1001/jama.1997.03540250033028
25. Nicklas BJ, Chmelo E, Delbono O, Carr JJ, Lyles MF, Marsh AP. Effects of resistance training with and without caloric restriction on physical function and mobility in overweight and obese older adults: a randomized controlled trial. *Am J Clin Nutr*. 2015;101:991-999. doi: 10.3945/ajcn.114.105270
26. Barbat-Artigas S, Rolland Y, Cesari M, Abellan van Kan G, Vellas B, Aubertin-Leheudre M. Clinical Relevance of Different Muscle Strength Indexes and Functional Impairment in Women Aged 75 Years and Older. *The Journals of Gerontology: Series A*. 2012;68:811-819. doi: 10.1093/gerona/gls254
27. Volaklis KA, Halle M, Meisinger C. Muscular strength as a strong predictor of mortality: A narrative review. *European Journal of Internal Medicine*. 2015;26:303-310. doi: https://doi.org/10.1016/j.ejim.2015.04.013
28. Cava E, Yeat NC, Mittendorfer B. Preserving Healthy Muscle during Weight Loss. *Adv Nutr*. 2017;8:511-519. doi: 10.3945/an.116.014506
29. Benjamini Y, Hochberg Y. Controlling the false discovery rate: a practical and powerful approach to multiple testing. *Journal of the Royal statistical society: series B (Methodological)*. 1995;57:289-300.
30. Garber CE, Blissmer B, Deschenes MR, Franklin BA, Lamonte MJ, Lee IM, Nieman DC, Swain DP. American College of Sports Medicine position stand. Quantity and quality of exercise for developing and maintaining cardiorespiratory, musculoskeletal, and

neuromotor fitness in apparently healthy adults: guidance for prescribing exercise. *Med Sci Sports Exerc*. 2011;43:1334-1359. doi: 10.1249/MSS.0b013e318213fefb
31. Balady GJ, Arena R, Sietsema K, Myers J, Coke L, Fletcher GF, Forman D, Franklin B, Guazzi M, Gulati M, et al. Clinician's Guide to Cardiopulmonary Exercise Testing in Adults. *Circulation*. 2010;122:191-225. doi: doi:10.1161/CIR.0b013e3181e52e69
32. Osman AF, Mehra MR, Lavie CJ, Nunez E, Milani RV. The incremental prognostic importance of body fat adjusted peak oxygen consumption in chronic heart failure. *J Am Coll Cardiol*. 2000;36:2126-2131. doi: 10.1016/s0735-1097(00)00985-2
33. Savonen K, Krachler B, Hassinen M, Komulainen P, Kiviniemi V, Lakka TA, Rauramaa R. The current standard measure of cardiorespiratory fitness introduces confounding by body mass: the DR's EXTRA study. *International Journal of Obesity*. 2012;36:1135-1140. doi: 10.1038/ijo.2011.212
34. Krachler B, Savonen K, Komulainen P, Hassinen M, Lakka TA, Rauramaa R. Cardiopulmonary fitness is a function of lean mass, not total body weight: The DR's EXTRA study. *European Journal of Preventive Cardiology*. 2020;22:1171-1179. doi: 10.1177/2047487314557962
35. Lolli L, Batterham AM, Weston KL, Atkinson G. Size Exponents for Scaling Maximal Oxygen Uptake in Over 6500 Humans: A Systematic Review and Meta-Analysis. *Sports Medicine*. 2017;47:1405-1419. doi: 10.1007/s40279-016-0655-1
36. Villareal DT, Aguirre L, Gurney AB, Waters DL, Sinacore DR, Colombo E, Armamento-Villareal R, Qualls C. Aerobic or Resistance Exercise, or Both, in Dieting Obese Older Adults. *New England Journal of Medicine*. 2017;376:1943-1955. doi: 10.1056/nejmoa1616338
37. Fitzmaurice GM, Laird NM, Ware JH. *Applied longitudinal analysis*. John Wiley & Sons; 2012.
38. Coffman CJ, Edelman D, Woolson RF. To condition or not condition? Analysing 'change' in longitudinal randomised controlled trials. *BMJ Open*. 2016;6:e013096. doi: 10.1136/bmjopen-2016-013096
